# ATP-competitive inhibitors for cancer treatment – kinases and the world beyond

**DOI:** 10.1039/d5md00235d

**Published:** 2025-06-25

**Authors:** Ana Jug, Janez Ilaš

**Affiliations:** a University of Ljubljana, Faculty of Pharmacy Ljubljana Slovenia janez.ilas@ffa.uni-lj.si

## Abstract

Adenosine 5′-(tetrahydrogen triphosphate) (ATP), an essential molecule for cellular energy transfer, plays a crucial role in various biochemical processes, including protein folding, DNA repair and intracellular signalling. A promising strategy for the development of anticancer therapies is to target ATP-binding sites of proteins involved in these processes with ATP-competitive inhibitors. They either mimic ATP to block its binding or bind allosterically to induce conformational changes that prevent ATP interaction. While protein kinases are the main focus of ATP-competitive inhibitors used in cancer therapy, other non-kinase targets such as Hsp90, Topo II, p97, RNA helicases and ABC transporters are also recognized as important molecular targets. Their inhibition can overcome resistance to kinase inhibitors, which develops due to mutations in kinase domains, and at the same time alter essential properties of cancer cells. Although they target different protein families, selectivity remains a challenge due to the conserved nature of ATP binding sites. However, the structural differences between the target proteins allow the development of specific inhibitors. In addition, dual inhibitors targeting multiple ATP-dependent proteins can increase therapeutic efficacy, reduce drug resistance and minimize side effects. Several ATP-competitive kinase inhibitors are already approved for clinical use and many more are in clinical trials, demonstrating their potential in cancer therapy. In this review, we focus on ATP-competitive inhibition in cancer therapy beyond kinases, highlighting recent advances and challenges in the field while applying lessons learned from the development of kinase inhibitors.

## Abstract 2

This review offers a detailed examination of small-molecule ATP-competitive inhibitors, placing particular emphasis on their role in targeting non-kinase proteins involved in the initiation and progression of cancer.

## Graphical abstract

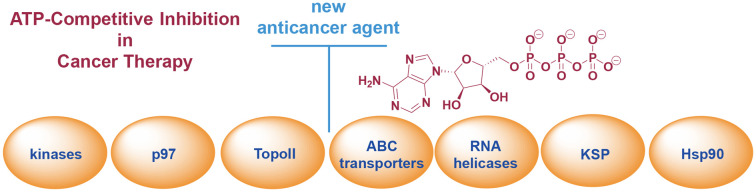

## 1. Introduction

As one of the main causes of premature mortality, cancer is one of the diseases with the greatest health burden in the modern world. In 2020, there were 20 million newly observed cases and almost 9.7 million deaths due to cancer.^1^ It is a very complex disease that consists of more than 200 pathologies and is not yet understood at all stages of its development, making it difficult to treat. One of the major drawbacks of conventional therapies such as chemotherapy is the development of drug resistance and severe adverse side effects. Significant progress has been made in recent years, *e.g.* in targeted therapy, stem cell therapy, nanoparticles, ablation therapy and radionics, but there is still much to be done in the field of cancer treatment.^2^

Adenosine 5′-(tetrahydrogen triphosphate) (ATP) is required for a large number of biochemical reactions. Its enzymatic cleavage at the terminal phosphate group releases stored chemical energy, which is the foundation for the proper functioning of living organisms. ATP has principal role in human biochemistry and bioenergetics.^3^ An ATP molecule consists of adenine, a ribose sugar and three phosphate groups linked in sequence by phosphodiester bonds. Electronegative charges create a repulsive force between the phosphate groups, so that a considerable amount of energy is stored in the phosphate–phosphate bonds. Once an ATP molecule is hydrolysed into adenosine diphosphate (ADP) and a free phosphate group, the stored energy is released and used for cellular processes such as active transport, protein synthesis, intracellular signalling and metabolism.^4^

**Fig. 1:**
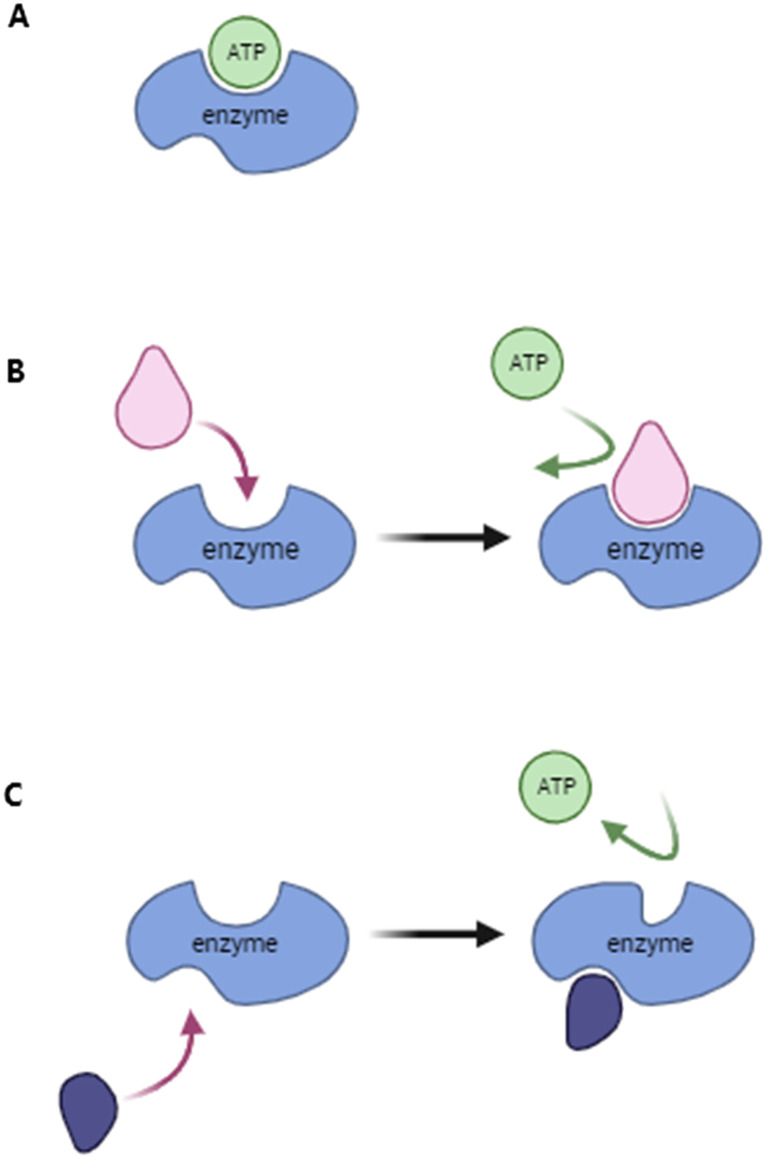
Different inhibition mechanisms. 1A: Active enzyme with bound ATP molecule. 1B: Competitive inhibitor binds to the ATP-binding pocket and prevents the binding of ATP to the enzyme. 1C: Allosteric inhibitor changes the conformation of the ATP-binding pocket and prevents binding.

Proteins such as kinases use ATP as an energy source for the transmission of information through cellular signalling cascades. Molecular chaperones (members of the HSP family) utilise ATP hydrolysis during protein translation to facilitate proper folding.^4^ Topoisomerase II enzymes require ATP to generate DNA breaks and to handle DNA supercoils and tangles.^5^ RNA/DNA helicases catalyse the separation of double-stranded RNA/DNA during their metabolism.^6^ p97 AAA ATPase is involved in many cellular processes and signalling pathways and uses ATP hydrolysis as a driving force.^7^ ATP-binding cassette transporters translocate substrates across membranes and their activity is powered by ATP.^8^ Since the above cellular processes are dysregulated in cancer, a change in the activity of the proteins involved would alter cancer pathology. In order to function, they utilise all the chemical energy released during ATP hydrolysis. Therefore, ligands that bind to the ATP-binding pocket or allosterically alter its conformation (Fig. 1) could be promising therapeutics. One problem lies in the high cellular ATP concentrations, especially in cancer cells as they are fast-proliferating and thus have higher energy demands for DNA replication and protein synthesis. Compounds that compete with ATP for its binding site must have a high affinity for their target, typically nanomolar affinity or lower to overcome high ATP intracellular levels. This is especially important for reversible inhibitors as their ability to modify the target depends on established equilibrium between free enzyme and enzyme-inhibitor complex. In an environment with high ATP concentration, equilibrium is shifted towards binding of ATP, reducing inhibitor's effectiveness.^9^ Consequently, inhibitors with high affinity must be designed which presents a challenging problem. ATP binding pockets are similar across different proteins which can increase off-target effects and severe toxicities. Nevertheless, there are some structural differences between mentioned enzymes that allow the development of selective inhibitors.^10^ Ultimately, the development and clinical success of imatinib was a breakthrough achievement in the development of ATP-competitive compounds for cancer treatment.^11^ This review describes the molecular features of ATP-competitive ligands used in cancer therapy and the molecular features required to achieve selectivity for the targets of interest, with a special focus on non-kinase inhibitors.

## 2. ATP-competitive protein kinase inhibitors for cancer treatment

Protein kinases are a large family of enzymes that regulate cell biology by covalently attaching a phosphate group to other target proteins. Phosphorylation leads to changes in the function of the target protein, its location or its association with other proteins. Protein kinases control the majority of signal transduction in our cells as well as other processes such as metabolism, cell cycle progression, cell differentiation and movement, transcription and apoptosis. Analysis of the human genome has shown that we have 518 protein kinase genes, which accounts for about 2% of all human genes.^12^ Due to their large number and their involvement in essential cellular processes, their dysregulation often leads to the development of cancer.

**Fig. 2:**
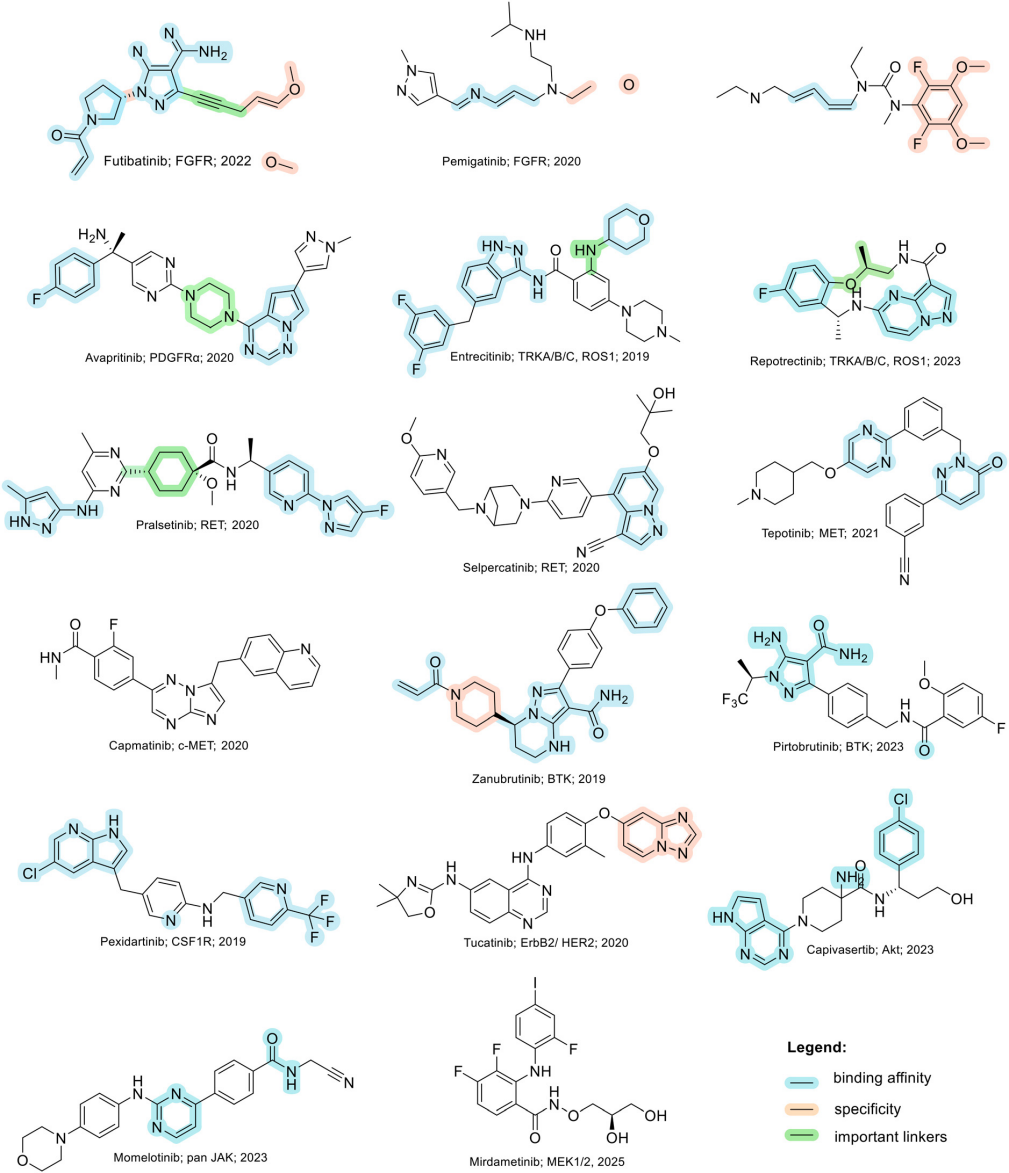
Structures of FDA approved ATP-competitive protein kinase inhibitors since 2019 (name; molecular target; year of approval).

There are 85 small molecule protein kinase inhibitors approved by the FDA for the treatment of various cancers, with the most recent approval of mirdametinib on February 11, 2025.^13^ Although this review highlights the development of non-kinase inhibitors, scaffolds are often inspired by kinase inhibitors due to similarities in binding sites. Libraries of kinase inhibitors are used to generate initial hits that are later optimized to specifically match the ATP sites of individual non-kinase proteins. Strategies for lead structure optimization are sometimes also inspired by kinase approaches. It has been established that in order to design ATP-mimetics, phosphate groups need to be removed from the structure as they decrease bioavailability and stability of kinase inhibitors. But as the phosphate groups make important interactions in the phosphate-binding pocket that contribute to ATP's binding energy, they need to be replaced in order to retain potency. If H-bond pattern of adenine is maintained and hydrophobic pockets that are not occupied by ATP are filled by new substituents, compounds with decreased IC_50_ values can be obtained.^14^ It is important to understand protein kinase inhibitors and their binding mechanisms in order to develop ATP-competitive non-kinase inhibitors. This section provides a brief description of protein kinase inhibitors that are ATP-competitive and have been approved by the FDA since 2019. Fig. 2 shows their structures and highlights the key functional groups that contribute to achieving potency and selectivity, which are also a major obstacle in the development of non-kinase ATP-competitive inhibitors.

Futibatinib is a covalent pan-fibroblast growth factor receptor (FGFR) inhibitor. The acrylamide moiety binds covalently to Cys491, which is located in the glycine-rich loop. Since this loop is very flexible and well conserved in protein kinases, the rigid linker is of great importance for the covalent binding of the molecule to the cysteine. The analysis showed that rigid, small cyclic structures work best. The pyrrolopyrimidine moiety mimics the adenine part of the ATP molecule, while the pyrrolidine overlaps with the ribose part. Selectivity for FGFR over the epidermal growth factor receptor (EGFR) can be achieved by changing the stereochemistry of the pyrrolidine ring at position 3′. *R*-Pyrrolidine analogues inhibit EGFR better, while *S*-pyrrolidine analogues inhibit FGFR better. By changing the stereochemistry, the orientation of the warhead is altered, leading to the formation of covalent bonds with cysteine residues at different positions in the ATP binding pocket. Selectivity for FGRF is further enhanced by the introduction of a 3,5-dimethoxybenzene ring that fills the unique hydrophobic pocket in the ATP binding site. A straight-chain alkyne linker is necessary to properly position the 3,5-dimethoxybenzene moiety to reach the binding pocket.^15^ Crystal structure of futibatinib in covalent complex with FGFR1 has been resolved (PDB: 6MZW).

Pemigatinib, like futibatinib, is a pan-FGRF inhibitor. However, pemigatinib acts as a potent non-covalent inhibitor, due to a network of van der Waals and hydrogen bond interactions (PDB: 7WCL). The pyrrolopyridine moiety mimics adenine and forms two hydrogen bonds with the hinge region in the ATP binding site. The difluoromethoxyphenyl ring occupies the hydrophobic pocket in a similar manner to the 3,5-dimethoxybenzene ring of futibatinib. A methoxy oxygen forms an additional hydrogen bond in the hydrophobic pocket. The solubilizing morpholine group is exposed to the solvent and does not form specific interactions.^16^

*Erdafitinib* is another FDA-approved FGRF inhibitor that has a similar binding mode to pemigatinib.^17^ Selectivity to FGRF is achieved by a 3,5-dimethoxybenzene moiety, as with the previously described drugs. However, the potency of *erdafitinib* (IC_50_ = 1.2 nM)^18^ is lower compared to pemigatinib (IC_50_ = 0.2 nM).^16^ As mentioned above, the tricyclic scaffold of pemigatinib forms two hydrogen bonds with the amino acid residues in the hinge region, whereas the quinoxaline core of erdafitinib forms only one hydrogen bond. More hydrogen bonds stabilize the conformation of pemigatinib, so that its efficacy against FGRF is higher compared to pemigatinib. The methylpyrazole group of *erdafitinib* is used as a solubilizing group and, similar to the morpholine group in pemigatinib, does not interact with the binding site (PDB: 5EW8).

Avapritinib acts as an inhibitor of the platelet-derived growth factor receptor α (PDGFRA) and is currently the only approved drug for the treatment of mutated gastrointestinal stromal tumors (GIST). It was discovered that upon binding, a new pocket appears to form in the ATP-binding site of PDGFRA (between the αC helix and the Gly-rich loop, hence the name Gα). There are several crystal structures of avapritinib and its derivatives in complex with wild-type or mutant KIT and PDGFRA (8PQ9, 8PQA, 8PQB, 8PQC, 8PQD, 8PQE, 8PQF, 8PQG, 8PQH, 8PQI, 8PQJ, 8PQK). The fluorobenzene moiety of avapritinib fills this pocket perfectly and forms an additional cation-π interaction within the Gα-pocket. To date, there are no other ligands that target the Gα-pocket, although it appears that their targeting is essential for achieving high efficacy. When replacing the piperazine linker with other heterocyclic residues, the binding affinity decreases significantly, although piperazine does not interact with the binding site in any appreciable way. This suggests that the piperazine linker is responsible for stabilizing the conformation of avapritinib and allowing the fluorobenzene to reach the Gα-pocket. The primary amine in the stereocenter forms an ionic interaction with the DFG motif. It has been shown that modifications to this amine are well tolerated.^19^

Entrecitinib was discovered as part of an HTS campaign to reduce the recurrence of cancer in patients treated with other anaplastic lymphoma kinase (ALK) inhibitors. The tetrahydropyranyl ring, used as a sugar-like substituent, has been shown to adequately fill the ribose-binding pocket in the ATP-binding site and improve binding affinity due to its rigid structure. The –NH linker at this position is important as it forms an intramolecular hydrogen bond with the adjacent carbonyl and stabilizes the bioactive conformation of entrecitinib. The aminoindazole moiety is responsible for the interactions with the hinge region, while the 3,5-difluorobenzyl is stacked between the glycine-rich loop and forms additional multipolar interactions with the interior of the pocket (PDB: 5FTO).^20^

Repotrectinib is a proto-oncogene tyrosine-protein kinase (*ROS1*) and tropomyosin receptor tyrosine kinase (TRKA, TRKB and TRKC) inhibitor. It is a rigid, macrocyclic molecule that is designed to anchor in the adenine pocket of TRK ATP-binding site. The ethyl ether linker is important for its conformational rigidity, minimizing the entropy penalty upon binding. Fluorophenyl and pyrazolopyrimidine moieties form interactions with the hinge region (PDB: 7VKO). Compared to other TRK inhibitors, repotrectinib does not extend into the solvent area which can help overcome development of resistance mutations.^21,22^

Pralsetinib is a REarranged during transfection (RET) inhibitor that is used to treat various types of cancer such as lung cancer and thyroid cancer. Its amino–pyrazole moiety is able to form three hydrogen bonds with the hinge region in the ATP binding site. The hexane linker connects it to a pyridine–pyrazole moiety located in close proximity to the P-loop, where it forms a π–π interaction with amino acid residues of loop.^23,24^ When the stereochemistry of the linker is altered, a more than 100-fold reduction in the potency of the ligand is observed. This indicates the importance of the linker conformation, as it extends the pyridine–pyrazole unit deep into a binding pocket where it forms an additional hydrogen bond with lysine. In an attempt to restrict the linker conformation, the methoxy group and the amide were joined to form a spiro skeleton. Although the activity of this ligand against wild-type RET was twofold lower than that of pralsetinib, it showed higher selectivity against RET mutations (V804M and M918T mutations), which could minimize the development of resistance and improve patient outcomes.^25^

Selpercatinib is a RET inhibitor with a similar binding mode and clinical efficacy to pralsetinib.^26^ The nine-membered pyrazolo ring of selpercatinib occupies the adenosine binding pocket. The pyrazolo[1,5-*a*]pyridine ring and the hydroxymethyl group form van der Waals interactions with the side chain of Y806. The hydroxymethyl group protrudes through the solvent front at the opening of the adenosine binding pocket. A solved crystal structure of selpercatinib bound to RET (PDB: 7JU6) and a RET–pralsetinib complex (PDB: 7JU5) allow a detailed comparison of the binding modes and provide information on the contributions of the individual functional groups to the binding affinity.

Tepotinib binds to the mesenchymal–epithelial transition factor (c-MET) kinase in a U-type binding mode, similar to all class I MET kinase inhibitors.^27^ Pyrimidine and pyridazine rings help to maintain this U-binding mode and form hydrogen bonds with the hinge region of the ATP binding site. The carbonyl group attached to the pyridazine forms an additional H-bond interaction. The molecule is also capable of forming π–π stacking interactions. Crystallographic data of tepotinib in complex with c-MET mutants are available (PDB: 8AU3, 81U5 and 8AW1). In an effort to improve efficacy and minimise side effects, Yao *et al.* introduced two chiral centres into tepotinib by adding two methyl groups, one at each CH_2_ linking carbon. Only the molecule with (*R*, *S*) configuration (IC_50_ = 1.6 nM) was able to outperform the binding activity of tepotinib (IC_50_ = 4 nM). The increase in binding activity is possible because the methyl group forming the chiral C2 centre provides an additional π–π stacking interaction that enhances the inhibitory activity. In addition, *N*-methylpiperazine points in a different direction compared to tepotinib.^28^

Capmatinib^29^ is a potent ATP-competitive and reversible MET inhibitor with an average IC_50_ of 0.13 nM. It exhibits high selectivity as a c-MET kinase inhibitor, and is inactive against RONβ, another member of the c-MET receptor tyrosine kinase (RTK) family, and is as well inactive against EGFR and HER-3, which belong to the EGFR RTK family.^30^ Although no detailed structural data on the binding mode of capmatinib are available, a wealth of clinical information has been reported.^31–33^

Zanubrutinib is an irreversible Bruton's tyrosine kinase (BTK) inhibitor. In order for the acrylamide moiety to form a covalent bond with Cys481, the molecule must be in an *S*-configuration. The cyclic core with the attached amide group forms three hydrogen bonds with the amino acid residues in the hinge region and the nitrogen atom on the pyrazole moiety forms an additional water bridge. The terminal phenyl group is responsible for a π–π stacking interaction (PDB: 6J6M). Zanubrutinib exhibits high selectivity towards other protein kinases, which is probably due to its intrinsic structural selectivity. However, if the piperidine linker is replaced by aromatic rings, the on-target activity is retained, but the selectivity for BTK over EGFR is lost.^23,34^ Mutations in C481S prevent irreversible inhibitors to form a covalent bond with the binding site, resulting in drug resistance. Great efforts have been made to develop new, reversible BTK inhibitors.^35^

Pirtobrutinib was developed as a reversible BTK inhibitor. The interactions formed are similar to those of zanubrutinib. Pirtobrutinib also forms three hydrogen bonds with the hinge region. It forms two water-mediated hydrogen bonds and a π–π stacking interaction. However, pirtobrutinib does not interact with Cys481 and it extends further towards the C-helix and activation loop compared to zanubrutinib (PDB: 8FLL).^36^

Pexidartinib is a colony-stimulating factor 1 receptor (CSF1R) kinase inhibitor. It has been approved by the FDA but not by the EMA due to concerns regarding its benefit–risk balance. The therapy shows a good response in patients with tenosynovial giant cell tumour, however, hepatotoxicity is a known risk and should be considered.^37^ Pexidartinib is a potent CSF-1R inhibitor (IC_50_ = 2.1 nM). The nitrogen atoms on the pyridine–imidazole backbone form two hydrogen bonds with the hinge region in the ATP binding site. Another important hydrogen bond with the DGF motif is formed by the nitrogen at the center pyridine. Trifluoromethyl-substituted pyridine extends into the hydrophobic binding pocket and forms π–π stacking interactions, while chloro-substituted pyridine forms another π–π stacking interaction (PDB: 4R7H).^38,39^

*Tucatinib* is an ATP-competitive and reversible inhibitor of human epidermal growth factor receptor-2 (HER2) that exhibits over 500-fold selectivity over HER4 and EGFR.^40^ Its pyridine-triazolo moiety forms a key hydrogen bond with HER-2 specific Ser783 in the ATP binding site, resulting in selectivity for HER2.^41^ Following tucatinib, other selective HER2 agents have been developed that interact with Ser783 and also stimulate proteasomal degradation of the receptor. The dual mechanism is a promising way to increase the efficacy of HER2 inhibitors.^42^

Capivasertib is a protein kinase B (PKB or Akt) inhibitor. Its pyrrolopyrimidine ring is responsible for two hydrogen bond interactions with the hinge region. *p*-Chlorophenyl group is pushed into a hydrophobic pocket and basic amino group reaches into the acidic hole between Glu234 and Glu278 due to an axial conformation of the central piperidine ring. The basic amino group also interacts with the sulfur of Met281 and forms three hydrogen bonds. NH amide and hydroxyethyl do not form interactions with the protein itself, but the hydroxyethyl chain is directed to a solvated region where it can form water-mediated interactions with the side chain (PDB: 4GV1).^43^

Momelotinib is Janus kinase (JAK1, JAK2 and JAK3) inhibitor. The crystal structure of JAK2 in complex with momelotinib (PDB: 8BXH) and momelotinib docked to the JAK3 ATP-binding site show that the pyrimidine ring and the 2-amino NH each form a hydrogen bond with the hinge region each. The phenylamide group forms an additional hydrogen bond, while the morpholine is exposed to the solvent and does not form any significant interactions.^44,45^ The introduction of a fluorine substituent at position 5 of the pyrimidine ring improved potency against all three JAK targets, in addition to improved pharmacokinetic properties compared to momelotinib.^45^

Although no new epidermal growth factor receptor tyrosine kinase inhibitors (EGFR-TKIs) have been approved by the FDA since 2019, research in this field continues. Since resistance to osimertinib, a third-generation EGFR-TKI approved by FDA in 2015 for treatment of non-small cell lung cancer (NSCLC) is observed, development of fourth-generation EGFR-TKIs is urgent to overcome C797S mutation in EGFR-TK causing resistance of third-generation EGFR-TKIs. Progress in this area of research was recently described by Das *et al.*^46^

## 3. ATP-competitive non-kinase inhibitors for cancer treatment

### 3.1. ATP-binding cassette transporters inhibitors

ATP-binding cassette (ABC) transporters are a large class of transporter membrane proteins which translocate a variety of different substrates across cell membranes using energy from ATP hydrolysis. While importers are only found in prokaryotes, exporters are present in both, prokaryotes and eukaryotes, 48 of them are found in humans. They are involved in the efflux of chemotherapeutics and the development of multidrug resistance and are overexpressed in cancer cells, so their inhibition could improve the treatment of cancer.^8,47^

To date, no inhibitors that bind to the nucleotide binding domain (NBD) have reached clinical trials. However, there is a lot of ongoing research done on this topic. For example, an NBD model based on P-glycoprotein structure was made (PDB: 4Q9H and 6C0V) and docking scores of ATP-mimetics were calculated to see if they could interact with the NBD. SN202, AICAR and ribavirin were shown to suppress ATPase activity and when incubated with paclitaxel enhance its effects on wild-type and paclitaxel-resistant cell lines.^48^

MK571 is an inhibitor of multidrug resistance-associated protein 1 (MRP1) which is well established for research purposes, but is not used in clinical use because of its poor pharmacokinetic properties and specificity. The reason for its poor specificity could be binding to the NBD that is well conserved amongst different ABC transporters.^49^

### 3.2. Kinesin spindle protein inhibitors

The kinesin spindle protein (KSP) or Eg5 is a mitotic motor protein that belongs to the kinesin superfamily. It acts during mitosis and transports vesicles, organelles and microtubules with energy from ATP hydrolysis. It forms a homotetrameric structure that is responsible for the spindle formation and function – it binds antiparallel microtubules and slides them apart. It is also responsible for centrosome separation and chromosome segregation. Inhibition of Eg5 leads to mitotic arrest and the formation of monoastral spindles, making it an attractive anticancer target.^50,51^

KSP inhibitors in clinical trials (http://ClinicalTrials.gov) such as ispinetib (ID: NCT00089973), filanesib (ID: NCT01372540, NCT02092922, NCT01372540), SB-743921 (ID: NCT00136513, NCT00343564), EMD544085 (ID: NCT00527684, NCT00527671, NCT00527677), MK-0731 (ID: NCT00104364), AZD4877 (ID: NCT00661609), ARQ 621 (ID: NCT00825487) and litrosenib (https://www.clinicaltrials.jp/ ID: JapicCTI-111496, 2523355-001, I1Y-MC-JFBF, I1Y-MC-JFBE) do not bind to the nucleotide binding pocket, but to an allosteric binding site located in loop 5 (L5) between α2 and α3 helices, 12 Å from the ATP-binding site. Upon binding they induce a conformational change in switch I and switch II loops that are responsible for coordination of ATP binding and hydrolysis (ispinesib–Eg5 complex PDB: 2IEH), preventing Eg5 movements along microtubules. Despite their ability to alter ATP's binding site, inhibitors that bind to the conventional L5/α2/α3 binding pocket are not ATP-competitive. A major problem with the inhibitors mentioned is the development of resistance to therapy due to mutations in the allosteric binding site,^52–54^ which is why a lot of work has been put into the development of new inhibitors that can circumvent resistance.

**Fig. 3:**
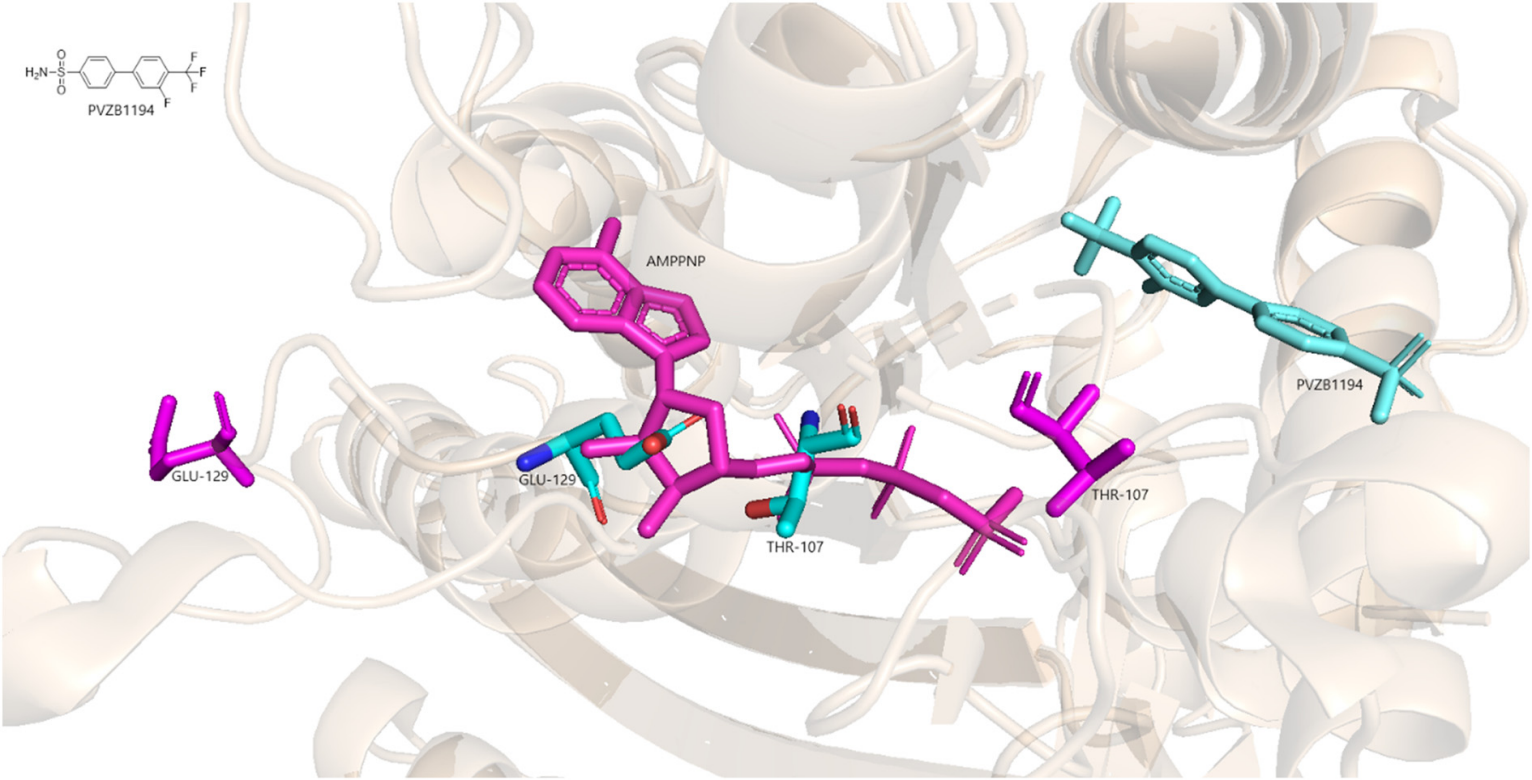
Superimposed Eg5 in complex with AMPPNP (PDB: 3HQD) and Eg5 in complex with PVZB1194 (PDB: 3WPN).

An important breakthrough was the discovery of the biaryl type Eg5 inhibitor PVZB1194, which binds to a different allosteric binding site between the α4 and α6 helices. It has been shown to alter the conformation of the ATP binding pocket, more specifically it pushes residues Thr107 and Glu129 into the pocket, resulting in insufficient space to accommodate an ATP molecule.^55,56^Fig. 3 shows superimposed structures of Eg5 in complex with a non-hydrolyzable ATP analogue, 5′-adenylyl-β,γ-imidodiphosphate (AMPPNP) (PDB: 3HQD) and Eg5 in complex with PVZB1194 (PDB: 3WPN). When PVZB1194 is bound to the protein, the blue coloured residues Thr107 and Glu129 are pushed into the ATP binding site and collide with AMPPNP. The pink-coloured residues represent Thr107 and Glu129 without an allosteric inhibitor bound, pointing away from the binding site and allowing nucleotide binding.

The features required for binding of the biphenyl-type inhibitors to the α4/α6 pocket include two aromatic rings, two H-bond donors and a hydrophobic feature. In the case of PVZB1194, the phenyl groups represent the aromatic ring features, the amide group is the H-bond donor and the trifluoromethyl group is the hydrophobic feature. In this position, groups larger than the trifluoromethyl group decrease the activity because they are too large and cannot fit into the α4/α6 binding pocket.^56^ Although PVZB1194 was initially thought to be an ATP-competitive inhibitor at first, kinetics analysis showed mixed non-competitive behaviour.^55^ However, other biaryl-type inhibitors were found to be ATP-competitive despite the fact that they bind to the same allosteric binding pocket.^52^ ATP-competitive carbazole-based inhibitors were later developed to improve the potency and physicochemical properties of PVZB1194. Here, the aniline provides flexibility to the two aryl groups and forms an H-bond with amino acid residues in the α4/α6 binding pocket. The trifluoromethyl group forms hydrophobic interactions and a π–π interaction. The lactam part of the molecule is responsible for an H-bond interaction and a π–π interaction.^57^ These changes led to increased IC_50_ values determined with ATPase assay compared to PVZB1194 (IC_50_ = 45 nM and IC_50_ = 120 nM, respectively).

### 3.3. p97 AAA+ (ATPases associated with various cellular activities) ATPase inhibitors

The human AAA+ ATPase p97 or valosin-containing protein (VCP) is a homohexameric enzyme involved in various cellular processes such as protein degradation, DNA repair and replication, NF-κB activation, cell cycle regulation, endoplasmic reticulum and mitochondria-associated degradation. In all these processes, it utilises the mechanical energy derived from ATP hydrolysis at the D2 domain to extract ubiquitinated proteins from large cellular structures such as macromolecular assemblies and lipid membranes. D1 domain exhibits lower ATPase activity and its primary role is mediation of hexamerization.^58^ Its involvement in such a variety of cellular processes and the fact that VCP is overexpressed in many cancer patients make it a very interesting target for cancer therapy, especially acute myeloid leukemia as it was shown that VCP is a key stress-related vulnerability in this disease.^59^

**Fig. 4:**
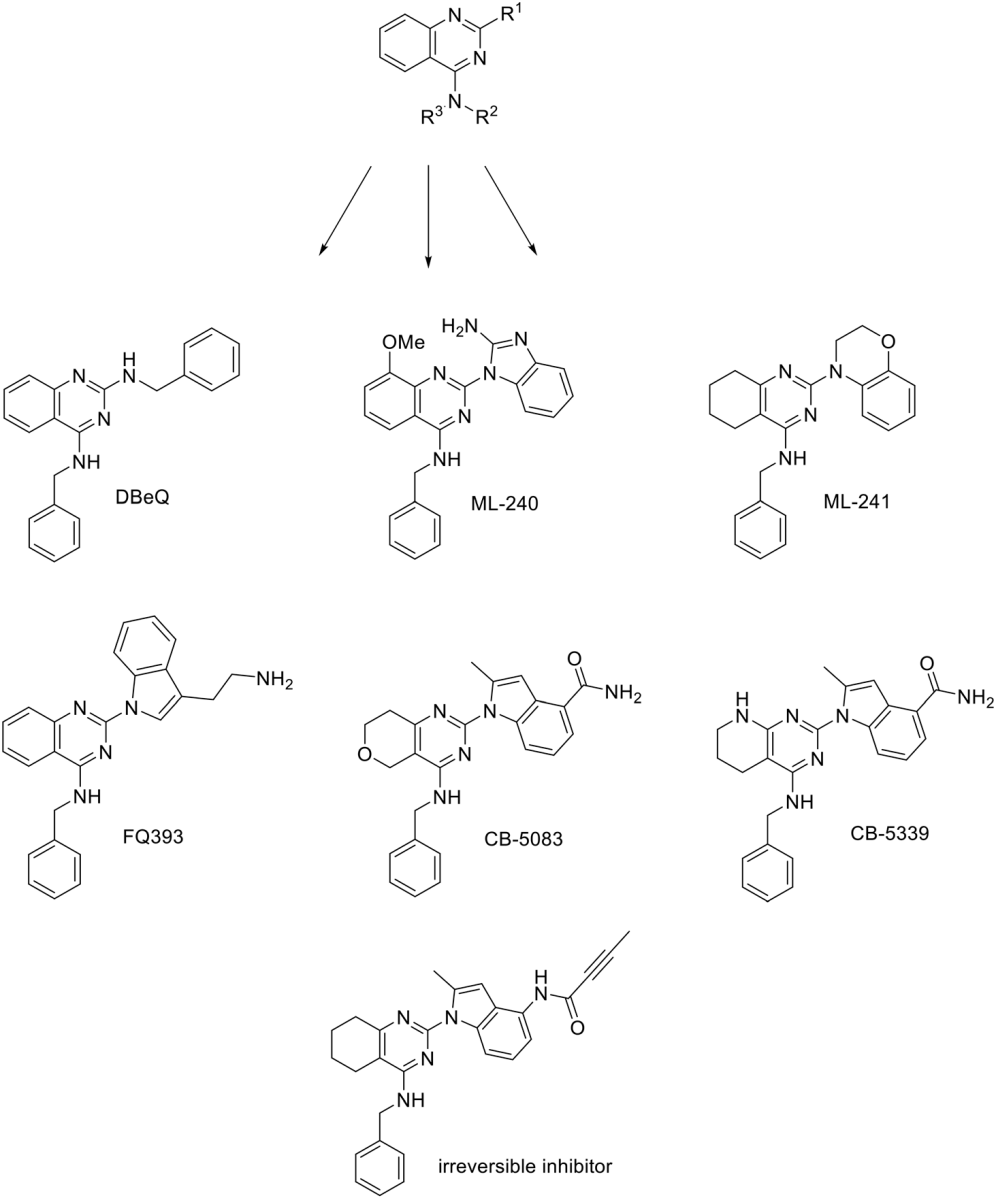
p97 inhibitors – quinazoline derivatives.

*N* ^2^,*N*^4^-Dibenzylquinazoline-2,4-diamine (DBeQ) was one of the first VCP inhibitors discovered through an HTS screening campaign.^60^ Since then, a lot of work has been done to improve its potency, specificity and pharmacokinetic properties that prevented it from entering clinical trials. SAR studies show that the benzyl group must be retained at the R^2^ position, while only one hydrogen atom is tolerated at the R^3^ position. The benzyl group fits into a small cavity that is typically occupied by the purine moiety of an ATP molecule. Variation of the substituents at the R^1^ position resulted in compounds ML240 with benzimidazole moiety and ML241 with benzoxazine moiety (Fig. 4). They inhibit ATPase activity with 100 nM IC_50_ values, whereas DBeQ inhibits it with 1.6 μM IC_50_ value. Although the quinazoline scaffold is present in many protein kinase inhibitors, kinase profiling showed selectivity of the tested compounds towards p97.^61,62^ ML240 and ML241 exhibit lower affinity for CNS targets compared to DBeQ (*K*_i_ < 10 μM for 1, 8 and 23 targets out of 43, respectively). Interestingly, ML240 and ML241 exhibit higher affinity for the D2 domain compared to D1 domain which opens up the possibility of selective modulation of p97's activity.^61,63^

By replacing benzylamine at R^1^ of DBeQ with tryptamine, a promising compound FQ393 was obtained.^64^ As with DBeQ, quinazoline, phenylmethanamine and amidogen form Van der Waals interactions with amino acid residues in the active pocket while quinazoline and indole of FQ393 form electrostatic interactions with the ATP binding site. The properties of FQ393 are not yet satisfactory, but the inhibitor is a good starting point for future modifications, especially due to its broad impact on cancer-related pathways shown by proteomic profiling.^64^ The replacement of quinazoline with 7,8-dihydro-5*H*-pyrano[4,3-*d*]pyrimidine, 2-amino on the benzimidazole ring with 2-methyl and the addition of an amido group on benzimidazole C5 resulted in the inhibitor CB-5083,^65^ which entered clinical trials in 2014 (http://ClinicalTrials.gov identifiers: NCT02223598 and NCT02243917). However, clinical trials were discontinued in phase I because the drug has an off-target effect on phosphodiesterase-6 and causes vision disturbance.^66^ A second generation VCP inhibitor, CB-5339 was designed to retain potency and minimize off-target effects. By replacing the pyranopyrimidine moiety of CB-5083 with pyridopyrimidine moiety, the activity of CB-5339 on phosphodiesterase-6 was reduced 15-fold compared to CB-5083. It was also found that VCP inhibition impairs DNA repair, so CB-5339 paired with standard chemotherapy could show synergistic effects.^59^ In 2020, CB-5339 entered clinical trials as a single agent in patients with relapsed or refractory acute myeloid leukemia (http://ClinicalTrials.gov identifier: NCT04402541).^67^ Since then, much research has been done to develop a new p97 inhibitor with an improved safety profile.^62,68–70^ This has been achieved by replacing the benzyl ring with pyrazine, which still fits into the purine cavity. The linker at the N4 position was extended by replacing the secondary amino group with hydrazine. The benzamido group at the N2 position was removed to minimize steric hindrance, and a smaller methoxy group was added as it works well in ATP-competitive kinase inhibitors.^62,71,72^

By replacing the amido group of CB-5083 with a Michael acceptor, an irreversible p97 inhibitor with low nanomolar concentration was obtained. Although the structures of the compounds are very similar, the covalent inhibitor is not ATP-competitive. Because of its irreversible binding it has an advantage over reversible inhibitors when competing with ATP. It also establishes a higher barrier for ATP to compete with as its *K*^app^_d_ value is considerably lower compared to endogenous ATP [*K*_d_(ATP) ∼ 2 μM, while *K*^app^_d_(inhibitor) = 33 ± 4 nM].^73^

Table 1 reports properties and current status of described p97 inhibitors such as IC_50_ values extracted form ATPase and cell-based assays, their progression to animal models and clinical trials and their pharmacokinetic properties where data is available.

**Table 1:** Status pf p97 inhibitors

Inhibitor	On-target IC_50_	Cell-based IC_50_	Preclinical/animal models	Pharmacokinetics	(Pre)clinical trials
DBeQ	1.6 μM (ref. 74)	0.24–6.9 μM (ref. 74)	—	—	Preclinical^74^
ML-240	100 nM	0.9 μM	Retinal degeneration models; no cancer animal models reported	—	Preclinical^75^
ML-241	100 nM	3.5 μM	Suckling mouse model of rotavirus infection; no cancer animal models reported^76^	—	Preclinical^75,77^
FQ393	0.9 μM (ref. 64)	1.37–10.8 μM (ref. 64)	—	—	Preclinical^64^
CB-5083	11 nM (ref. 65)	<1 μM (ref. 65)	Xenograft models: multiple myeloma, B-cell lymphoma, solid tumors^65^	Moderate oral bioavailability (41%)^65^	Phase I terminated due to adverse ocular effects^66^
				Good metabolic stability (mouse liver microsomal half-life = 102 min)^65^	
				Good Caco-2 permeability with negligible efflux^65^	
			Disruption of UPS^65^		
CB-5339	11 nM (ref. 59)	<1 μM (ref. 59)	Tumor-bearing pet dogs with naturally occurring cancers: Myeloma, lymphomas, solid tumors^78^	Oral bioavailability higher compared to CB-5083 (data not publicly available)	Phase I ongoing for acute myeloid leukemia and myelodysplastic syndrome^67^
				Poor metabolic stability (mouse liver microsomal half-life = 10–24 min)^79^	

DBeQ is mainly used as a tool compound to study the role of p97 *in vitro* due to its poor selectivity and pharmacokinetic properties such as solubility, low bioavailability and metabolic stability. It has been a starting point for the optimization of new p97 inhibitors. ML240 and ML241 share similar structure and show similar on-target activity.^75^ However, ML241 is less potent in cell-based assays*.* FQ393 is still in its early preclinical stage, but it exhibits moderate on-target activity and good antiproliferative activity in various cancer cell lines, making it a promising lead compound appropriate for further optimization. CB-5083 and CB-5339 are the most characterized and advanced p97 inhibitors. CB-5339 was made to diminish adverse effects caused by CB-5083. Its metabolic stability is low compared to CB-5083. Its short mouse liver microsomal half-life is attributed to sulphur atoms and pyridine moieties that act as metabolic liabilities. Nevertheless, CB-5339 has sufficient oral bioavailability and *in vivo* pharmacokinetics to support clinical development.^79^

### 3.4. RNA helicase inhibitors

RNA helicases are a large class of enzymes involved in RNA metabolism and eukaryotic cellular processes such as translation, transcription, ribosome assembly and RNA decay. There are more than 70 members, all of which consist of two RecA-like domains connected by a flexible linker. Based on sequence motifs, they are categorized into superfamily 1, which consists of the Upf1-like family, and superfamily 2, which is further clustered to DEAD-box, DEAH-box/RNA helicase A-like (DEAH/RHA), RIG-I-like and Ski2-like families. They all act as molecular motors, *i.e.* they use the energy gained from ATP hydrolysis to move along the RNA chain and remodel it. The use of ATP-competitive inhibitors could be an effective route to catalytic inhibition, but specificity for the aforementioned members must be achieved. This is a difficult task because the ATP-binding site is shared by all RNA helicases and many other ATP-dependent enzymes.^6,80^

Eukaryotic initiation factor 4A (eIF4A) is a member of the DEAD-box family and consists of eIF4A1, eIF4A2 and eIF4A3. They all function as helicases and are essential for ATP-dependent unwinding of double-stranded RNA.^81^ Elisabatin A, allolaurinterol^82^ and elatol are marine-derived inhibitors of eIF4A. Their discovery merely sets the stage for the discovery of more potent and successful ATP-competitive inhibitors.^83,84^ Since natural-derived compounds are difficult to synthesize, a novel indole-based small molecule elF4A inhibitor was discovered. The alkyl group at position 6 is required for binding activity and 2-carboxyl or another moiety with an acidic proton, *e.g.* tetrazole, is required for the formation of ionic interactions with the binding site. The NH hydrogen in the indole ring must be retained to enable H-bond formation. However, substituents at positions 4 and 7 can be advantageous. A Cl atom at position 4 improves the hydrophobic interactions with the binding site. It is assumed that the binding pocket around position 7 is too narrow for a Cl atom, but an F atom is tolerated. The described compounds show selectivity towards elF4A3 over elF4A1, elF4A2 and other helicases (IC_50_ value of 0.97 μM for elF4A3 and >100 μM for all other mentioned targets).^85^

A breakthrough was achieved in 2019 when the crystal structure of human elF4A1 in complex with AMPPNP, RNA and rocaglamide-A was solved in the active closed state (PDB: 5ZC9).^86^ However, in the inactive, open state, only the structure of full-length yeast elF4A1 exists (PDB: 1FUU). Additional difficulties arise from the fact that the structure of the human elF4A1 protein in complex with ATP has not yet been elucidated, which complicates the development of ATP-competitive elF4A1 inhibitors. However, it is hypothesized that ATP binds in the interdomain cleft where the adenine part of the molecule interacts with the N-terminal domain and the phosphate groups extend to the C-terminal domain, leading to a conformational change. The above-mentioned crystal structures were used to identify novel phenyl–piperazine-based inhibitors. They bind to the nucleotide binding site and stabilize elF4A in a closed state with low energy. The benzyl group is responsible for the π-stacking interactions with the binding site. The nitrogen in the piperazine ring forms interactions with two arginine residues in the binding site and additional substituents form H-bonds and water bridges with conserved motifs I and II, which are responsible for the binding of ribose-ATP. Despite being identified as hits in biochemical assays, most of phenyl–piperazine compounds did not exhibit cell cytotoxicity. No *in vitro* permeability assays were done, but QikProp calculations suggest poor permeability might be the problem.^87^

### 3.5. DNA topoisomerase II inhibitors

The need for double stranded DNA to unwind during transcription and replication leads to a topological problem. When one part of the molecule unwinds, another part of the DNA molecule must overwind to compensate. To prevent DNA overwinding topoisomerases introduce transient double stranded breaks in the DNA chains.^5^ Their involvement in DNA replication, transcription and chromosome segregation makes DNA topoisomerases attractive targets in cancer therapy.

Topoisomerase II inhibitors are divided into two groups based on their inhibition mechanism. The drugs in the first group, known as “Topo-II poisons”, act as stabilisers of the covalent Topo-II–DNA complexes. The complexes formed act as toxins that trigger cell apoptosis. The second group comprises of catalytic Topo II inhibitors, which inhibit the catalytic function of the enzyme without damaging the DNA. Since topoisomerase enzymes use energy from ATP hydrolysis to move the DNA chain along the enzyme, small molecules that act as non-hydrolyzable ATP analogues are a promising group of topoisomerase inhibitors.^88,89^ Although there are still no clinically approved Topo II catalytic inhibitors on the market, research in this field is making progress.^90,91^

**Fig. 5:**
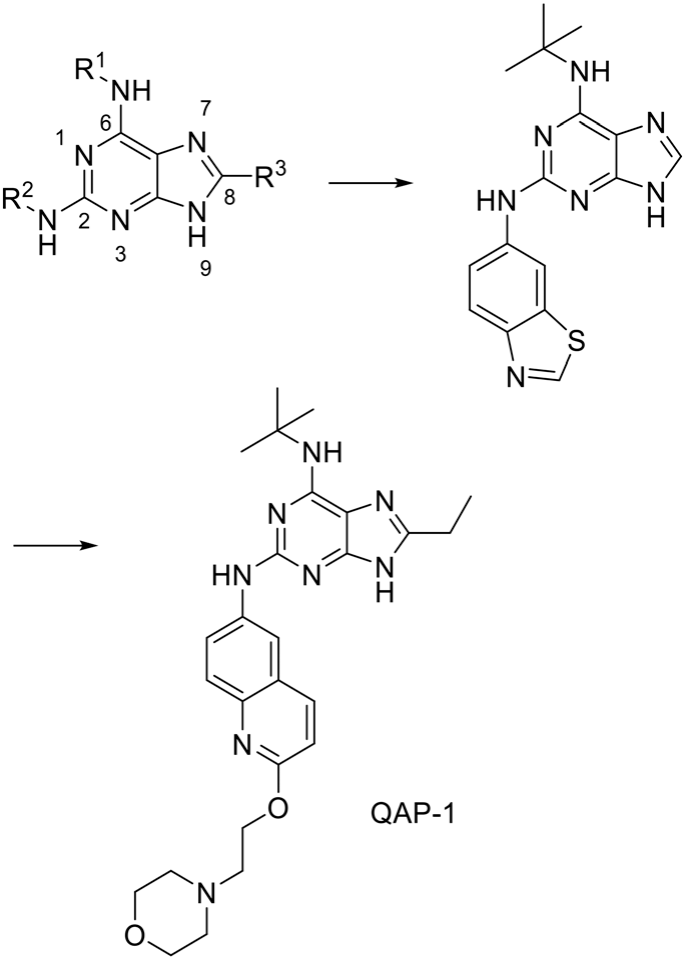
Design of QAP-1, the first catalytic ATP-competitive inhibitor of human topoisomerase IIα as a preclinical candidate.

A major breakthrough in the development of Topo II catalytic inhibitors was achieved in 2005 when the crystal structure of the ATP-ase domain was solved in complex with AMPPNP (PDB: 1ZXM). Since then, it has been successfully used to develop new ATP-competitive inhibitors.^91–98^ However, the first preclinical candidate, QAP-1, was developed based on the crystal structures of *Escherichia coli* DNA gyrase subunit B (PDB: 1EI1) and yeast topoisomerase II (PDB: 1PVG), as the crystal structure of human topoisomerase had not been solved at the time. By mimicking and adding additional interactions compared to those of ATP, the designed scaffold (Fig. 5) forms H-bonds with the same residues Asn120 and Asn91, but with a different purine ring orientation. This orientation allows the addition of substituents at positions 2 and 8 that can utilise new interactions not formed by ATP. With a small alkyl group at position 8, the displacement of water molecules from a nearby sub-pocket is possible and with a secondary amine at position 2, the formation of an H-bond and additional interactions with the adenine pocket are possible. NH linker at position 6 reaches the ribose binding pocket and forms hydrophobic interactions. The scaffold thus designed was used as a query to search the Novartis database where the hit (Fig. 5) was obtained. Its purine moiety forms the above-mentioned H-bonds and pushes the *tert*-butyl group into the ribose-binding pocket. The benzothiazole group is positioned at the entrance of the cavity, where it forms a π–π stacking interaction, and the thiazole nitrogen forms an additional H-bond with Ser149. Methylation of N9 nitrogen on the purine ring causes the inhibitory activity of the compound to be lost, confirming that N9 is involved in the formation of the key H-bond with Asn120. The ethyl group at position C6 and the replacement of the benzothiazole by a quinoline with an attached morpholino ethoxy group further improved the activity and yielded the first preclinical candidate, QAP-1.^99,100^ A study using 18-F labelled QAP-1 analogues implies they exhibit poor pharmacokinetic properties, especially fast clearance and low tumour uptake which does not make them useful without further optimization.^101^

**Fig. 6:**
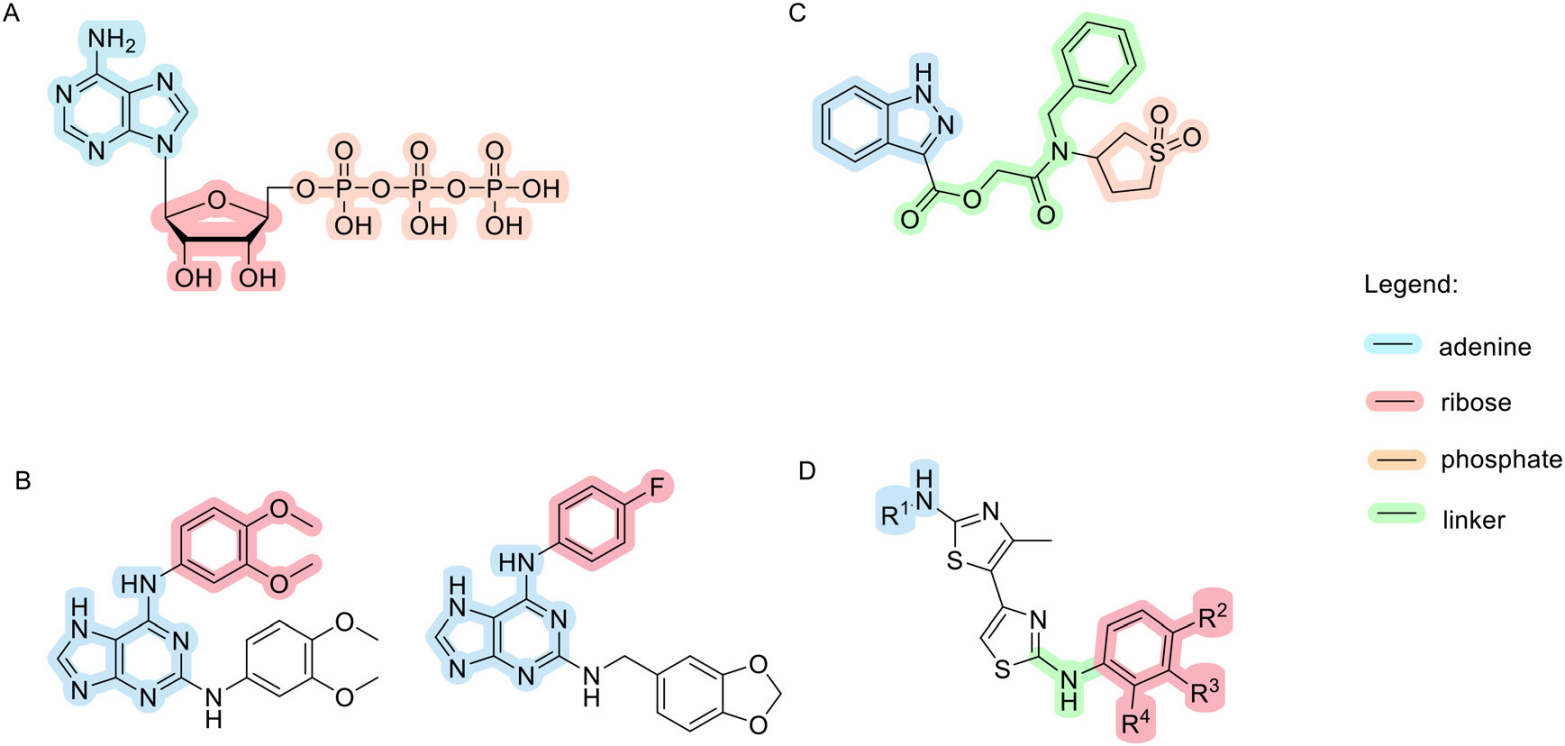
Chemical structures of Topo II inhibitors that act as ATP-mimetics compared to an ATP molecule.

A novel class of 1*H*-pyrazolo[3,4]pyrimidines and 9*H*-purines with inhibitory activity on Topo IIα has been identified, with prazolopyrimidine and purine mimicking the adenine moiety of ATP (Fig. 6A) and forming an H-bond with Asn120 in the binding site. Morpholine analogues at the C9 position mimic the ribose moiety and form interactions with the same amino acid residues as ATP. The structures of the two most promising compounds from this study are shown in Fig. 6B. Their IC_50_ values obtained on isolated enzyme are higher compared to those obtained in cell-based assays (left: 211 μM hTopoIIa and 36.2 MCF-7, right: 360 μM hTopoIIa and 45.9 μM MCF-7). These results imply that cell activity is a result of compounds inhibiting also other enzymes. Since they are ATP-competitive, they could bind to ATP binding sites of several protein kinases, but further studies need to be done to confirm this hypothesis.^102^ Later, a novel scaffold, 3-substituted 1*H*-indazole, was identified. The 1*H*-indazole ring and the sulfonyl group mimic the adenine ring and one of the phosphate groups, respectively. Flexible linkers allow the sulfonyl group to reach the binding pocket of the phosphate group, and large lipophilic substituents (*e.g.* a benzyl group) on the amide nitrogen of the linker enhance the inhibitory activity (Fig. 6C).^94^

Substituted 4,5′-bithiazoles are a new class of catalytic Topo II inhibitors. The first thiazole ring, which contains the N-terminal group, functions as a H-bond donor and mimics the purine ring of AMPPNP. The other end of the ligand reaches the AMPPNP phosphate group binding site and provides selectivity for human Topo II over bacterial DNA gyrase, as the R^2^ carboxyl group is oriented outside the phosphate binding pocket and forms H-bond interactions with other amino acid residues. Substituents on the benzene ring positioned in the spacious ATP–phosphate group binding pocket form similar interactions as the ribose sugar of ATP (Fig. 6D). Some substituted 4,5′-bithiazole analogues inhibit TopoII comparable to an approved TopoII poison etoposide (30–50 μM and 59.2 μM, respectively). Assay on cell lines confirm their activity with values 23.5–46.8 μM for HepG2 and 6.6–59.5 μM on MCF-7.^103^ Additional tests such as solubility, permeability, plasma protein binding *etc.* should be done to see if synthetized compounds exhibit potential for progression to animal models.

**Fig. 7:**
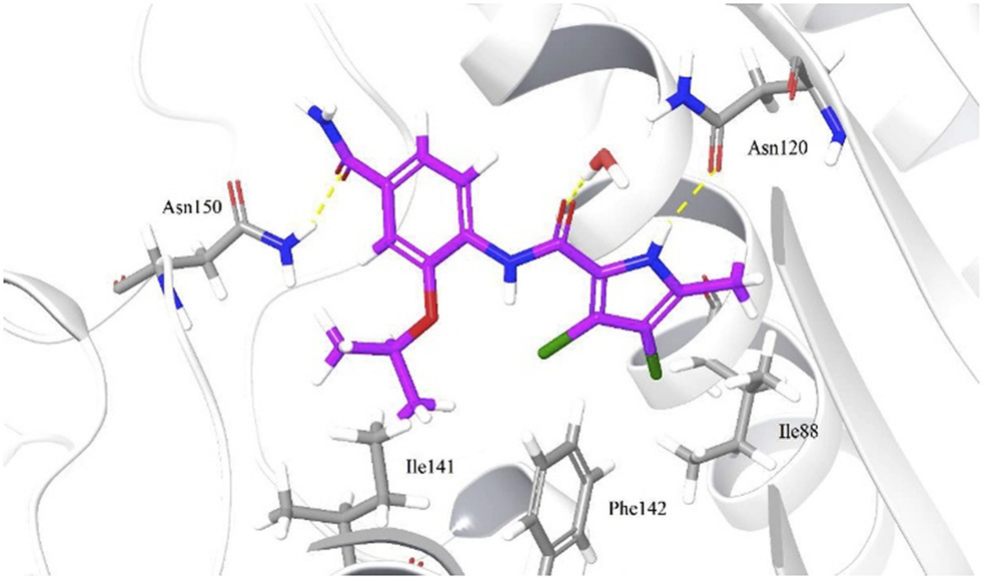
Predicted binding pose of 1,2,4-substituted *N*-phenylpyrrolamide inhibitor*.*^105^

1,2,4-Substituted *N*-phenylpyrrolamide inhibitors acting on human topoisomerase were discovered in the screening of highly potent bacterial topoisomerase inhibitors. The dichloromethylpyrrole moiety was found to be essential for the inhibitory activity, as it forms H-bonds with Asn120 and a conserved water molecule and occupies the hydrophobic pocket of the adenine binding site.^104,105^ If the substituent at position 4 is removed, the binding affinity is lost. If it is moved to position 5, the binding activity is reduced or lost. The substitution at position 4 is therefore essential for activity. The substituent at position 2 of the central benzene backbone is also required for binding activity. The isopropoxy group gave the best results,^104^ although in recent work benzoxy groups with different polar substituents were introduced to reach the phosphate binding pocket, which improved the binding affinity.^105^ Here, the introduction of basic centres (primary or secondary amines) improved cytotoxicity as well as antiproliferative activity. Ethylenediamine or its rigid cyclic derivatives such as piperidine performed best. Basic centres also improved the inhibitory activity at position 4. Some derivatives containing a carboxylic moiety were potent on-target, but not in cell-based assays which implies their poor permeability due to acidic nature. Fig. 7 shows the predicted binding pose of a 1,2,4-substituted *N*-phenylpyrrolamide inhibitor (2′ = isopropoxy, 4′ = amide) in the ATP-binding site of Topo II based on molecular docking. Overall, prepared compounds are highly active with IC_50_ values <1 μM on isolated target and HepG2/MCF-7 cell lines for the most potent ones. They are metabolically stable during phase I metabolism with majority observed half-lives >300 min. Thermodynamic aqueous solubilities are reasonable, between 2 and 20 μM. They need to be improved, but representative compounds still serve as promising leads for catalytic TopoII inhibition.^105^

**Fig. 8:**
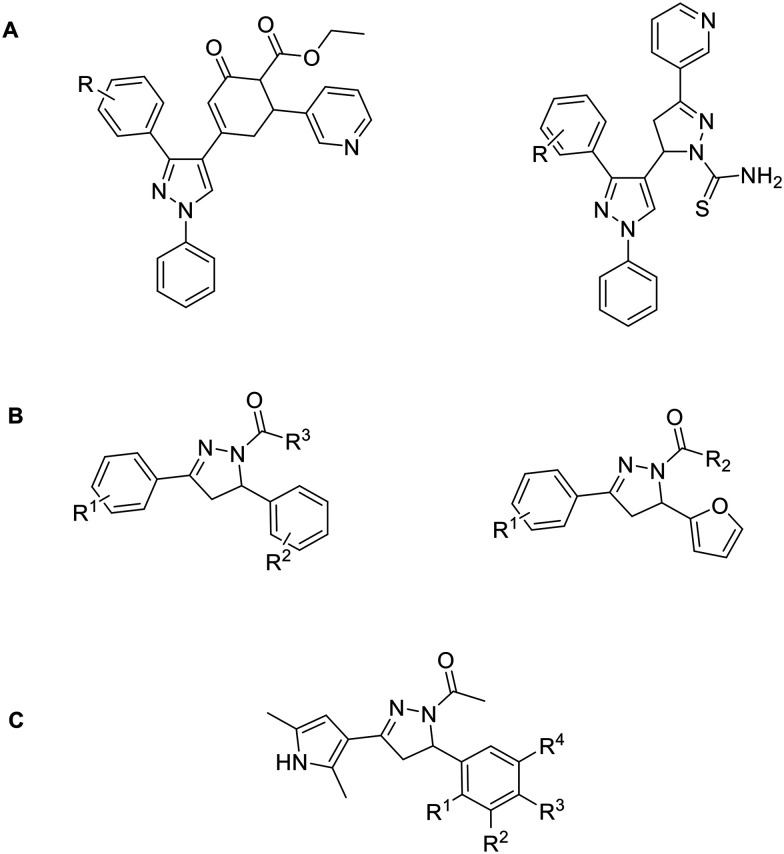
Topo II inhibitors bearing pyrazole and pyrazoline scaffolds.

Pyrazole derivatives, in particular ethyl 4-(3-(aryl)-1-phenyl-1*H*-pyrazol-4-yl)-2-oxo-6-(pyridin-3-yl)cyclohex-3-enecarboxylate and 5-(3-(4-fluorophenyl)-1-phenyl-1*H*-pyrazol-4-yl)-3-(pyridin-3-yl)-4,5-dihydropyrazole-1-carbothioamide derivatives are also known for their Topo II inhibition. Here, Br and Cl substituents on both *meta* and *para* positions inhibit Topo II activity, with the Br substituent on *meta* position of the benzene ring showing the best cytotoxic activity. It is hypothesised that the heterocyclic scaffold forms hydrophobic interactions, while ethyl ester oxygen and carbothioamide substituents form H-bonds with amino acid residues in the binding pocket (Fig. 8A).^92^

In the same year, an article was published on pyrazoline derivatives derived from chalcones and hydrazines (Fig. 8B). The most promising compound derived from chalcones has a benzo[*d*]thiazole ring at the R^3^ position, a 4-methoxy group at the R^2^ position and a 4-Cl substituent at the R^1^ position. The same 4-Cl substituent at the R^1^ position in a hydrazine-derived compound is thought to cause selectivity problems as it is the only compound from this series that inhibits not only Topo II but also Topo I.^106^ However, as both enzymes are involved in carcinogenesis, this observation could be exploited in the development of dual inhibitors to achieve synergistic anticancer effects and reduce drug resistance.^89^ A similar scaffold also inhibits Topo II (Fig. 8C), in which the nitrophenol ring extends into a phosphate binding pocket and the oxygen forms a hydrogen bond with amino acid residues (NO_2_ on R^1^ or R^3^ position). The hydroxyl group substituted on R^4^ of the same ring forms one hydrogen bond and a salt bridge with the magnesium ion. The dimethyl ring overlaps with the adenine part of the ATP molecule and the molecular scaffold is involved in the formation of hydrophobic interactions.^107^ Representative inhibitors bearing pyrazole and pyrazoline scaffolds showed significant cytotoxicity compared to etoposide on various cell lines. Their IC_50_ values are typically <10 μM, making them superior to previously mentioned 1*H*-pyrazolo[3,4]pyrimidines, 9*H*-purines and substituted 4,5′-bithiazoles in terms of *in vitro* cytotoxicity.

**Fig. 9:**
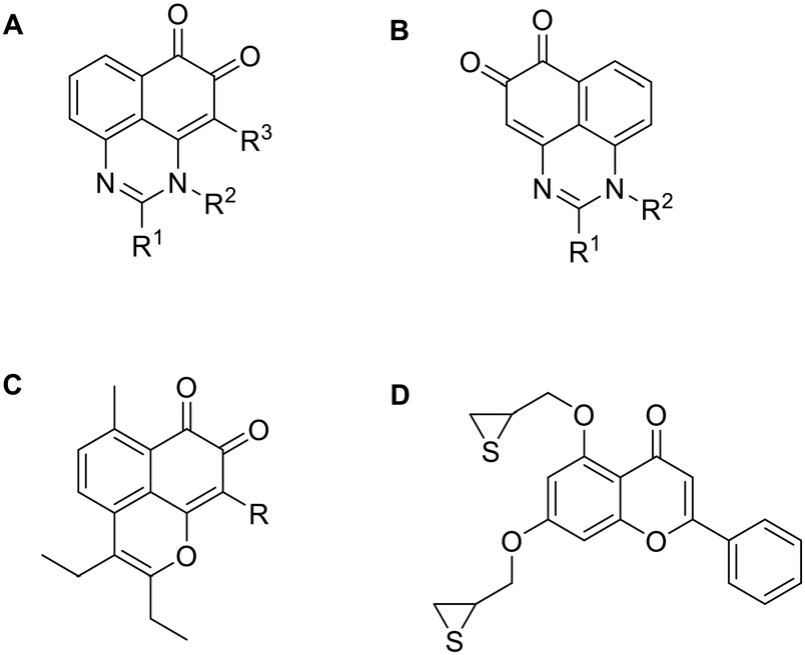
Topoisomerase inhibitors derived from natural products.

Compounds with *o*-quinone moiety such as mansonone^108^ and salvicine^109^ can alter topoII activity and exhibit antitumor properties (Fig. 9A and B).^96^ Compounds with *o*-quinone on ring A exhibit better cytotoxicity than those with *o*-quinone on ring B when R^1^ is substituted with an alkyl group. Substitution of R^1^ with an electron-donating aryl group reduces cytotoxicity, while an electron-withdrawing aryl group enhances it. Tests on Topo II poison resistant cell lines confirmed that the most potent compound in this series (R^1^ = *p*-chlorophenyl) does not act as Topo II poison. Although this was not confirmed, the results of the ATP-competition assay and docking indicate that it is an ATP-competitive catalytic inhibitor. The oxygen atoms at the C6 and C5 positions could form H-bonds with amino acid residues in the ATP-binding site and C2 phenyl could interact with Mg^2+^.^96^

SAR analysis of manosone F derivatives shows that the C9 substitution is crucial for cytotoxicity, with the 9-Br substituent increasing it the most. Flexible alkyl chains at C2 and C3 positions perform better than rigid aromatic rings, regardless of their length.^110^ It was confirmed that the most potent compound from the aforementioned study (Fig. 9C) acts neither as an intercalator nor as a Topo II poison. However, its activity is dependent on ATP concentration, suggesting that it may act as an ATP-competitive catalytic inhibitor. Further tests to confirm the mechanism of action need to be performed as it could also act as an allosteric inhibitor and not bind to ATP binding site.^111^

Chromone and xanthone derived compounds coupled with (thio)epoxide also showed inhibition and selectivity towards Topo II. The best results were obtained with 2-phenyl-chromenone core (Fig. 9D) which is placed into purine cavity where 2-phenyl ring forms hydrophobic interactions with the ATP binding site. The chromenone ring forms a π–π stacking interaction and a cation–π interaction with the Mg^2+^ cation which contributes to ATP-competitive mechanism of binding and greater selectivity for Topo IIα over Topo IIβ. Thioepoxide side chain at C3 position occupies the triphosphate binding pocket and forms two H-bonds. Such prepared compound inhibited Topo II at 20 μM concentration better than etoposide (33.8% and 27.5%, respectively). This chromone derivative was shown to act as a catalytic inhibitor rather than a Topo II poison, which might reduce the DNA-damage related side effects.^112^

### 3.6. Hsp90 inhibitors

Hsp90 is also being intensively studied as an individual anti-cancer target. It is a homodimeric chaperone protein involved in proteostasis under physiological and stress conditions. It assists in the proper folding of other proteins and their stabilization against heat stress and degradation. However, it also stabilizes proteins involved in tumorigenesis, making Hsp90 inhibitors attractive antitumor agents. The activity of Hsp90 is ATP-dependent, as it must undergo multiple conformations to function properly.^113^ The N-terminal ATP-binding pocket interacts only with the adenine and ribose part of the molecule, while the phosphates are turned outwards. Only when the N-terminal and the middle part of the protein associate, the γ-phosphate becomes buried. This circumstance enables the development of ATP-competitive Hsp90 inhibitors that bind to the ATP-binding pocket, stabilize the open protein conformation and preventing the activity of the protein.^114^ Hsp-90 has four isoforms: cytosolic Hsp90α and Hsp90β, endoplasmic reticulum Hsp90 (Grp94) and mitochondrial Hsp90 (TRAP1). N-terminal ATP binding site is highly conserved among all isoforms, so design of isoform-selective Hsp90 inhibitors is challenging. Nevertheless, research in this field is progressing rapidly. A review of isoform-selective and pan-Hsp90 inhibitors and the differences between the binding sites that enable their development has already been made,^115^ so this section describes only inhibitors that progressed to clinical trials or are somehow else important.

The original idea in the development of Hsp90 inhibitors was to develop pan-Hsp90 ATP-competitive inhibitors. Although many of them have been tested in clinical trials, no pan-Hsp90 inhibitor is in clinical use to date. They all lack efficacy and induce heat shock response (HSR) which is associated with various toxic effects. Examples of pan-Hsp90 inhibitors include antibiotic geldanamycin and its derivatives 17-AAG and 17-DMAG, resorcinol-based compounds such as ganetespib and onalespib, and the purine analogues PU-3 and PU-H71. 17-AAG entered clinical trials for leukemia, kidney cancer, melanoma and other malignancies, but they were terminated due to observed toxicity, low solubility, manufacturing difficulties and patent expiry.^116^ 17-DMAG has improved solubility and bioavailability compared to 17-AAG. It entered clinical trials for advanced solid tumours and refractory HER2+ cancers, but these were terminated due to toxicity and lack of commercial interests.^116^ Ganetespib entered clinical trials in combination with docetaxel for the treatment of non-small cell lung cancer, but failed to improve outcomes compared to docetaxel alone.^117,118^ Onalespib was tested as a single agent in patients with advanced solid tumours. Due to its lack of antitumor activity, onalespib is being tested in combination trials with paclitaxel (http://ClinicalTrials.gov Identifier: NCT02474173) and other anticancer agents or therapies.^119,120^ PU-H71 is currently in a phase I clinical trial as a single agent for patients with advanced malignancies (http://ClinicalTrials.gov identifier: NCT01393509)^121,122^ and in combination with ruxolitinib (http://ClinicalTrials.gov Identifier: NCT03935555).^123^ Another pan-Hsp90 inhibitor XL888 was assessed in combination with pembrolizumab and verumafenib in patients with melanoma and gastrointestinal adenocarcinomas (http://ClinicalTrials.gov Identifiers: NCT03095781 and NCT01657591). SNX-5422 is a prodrug which metabolizes to SNX-2112 which then binds into Hsp90 ATP binding site. It was evaluated in various clinical trials, but its development was discontinued due to unsuccessful results and financial troubles.^116^ Due to lack of success of pan-Hsp90 inhibitors, a new approach currently being used is design of isoform-selective Hsp90 inhibitors as they do not induce HSR. One way to selectively target mitochondrial TRAP1 is to add a positively charged triphenylphosphine (TPP) moiety that targets mitochondria. Examples are gamitrinib-TPP, a geldanamycin analogue and SMTIN-P01, a PU-H71 analogue. Gamitrinib-TPP is currently in a phase I clinical trial in advanced tumours (http://ClinicalTrials.gov identifier: NCT04827810). Only one class of Hsp90α selective inhibitors was discovered to date.^124^ They are based on structure of pan-inhibitor onalespib, but none of them has yet advanced out of preclinical evaluations. Luminespib is selective towards cytosolic Hsp90, but it inhibits Hsp90α and Hsp90β at similar rates. It was evaluated in many trials,^116^ but to date only pimitespib, which is also a Hsp90α and Hsp90β inhibitor is used in clinical practice. It is used in Japan for relapsed gastrointestinal stromal tumours.^125,126^

Although Hsp90 inhibitors have proven clinically ineffective in the past for many reasons, experts predict that advances in isoform-selective inhibitors offer a new opportunity for clinical success.^115^Table 2 lists Hsp90 inhibitors that progressed into clinical trials and their current status.

**Table 2:** Hsp90 inhibitors in clinical trials and their current status

Inhibitor	Structure	Target	Clinical trials	Status
17-AAG	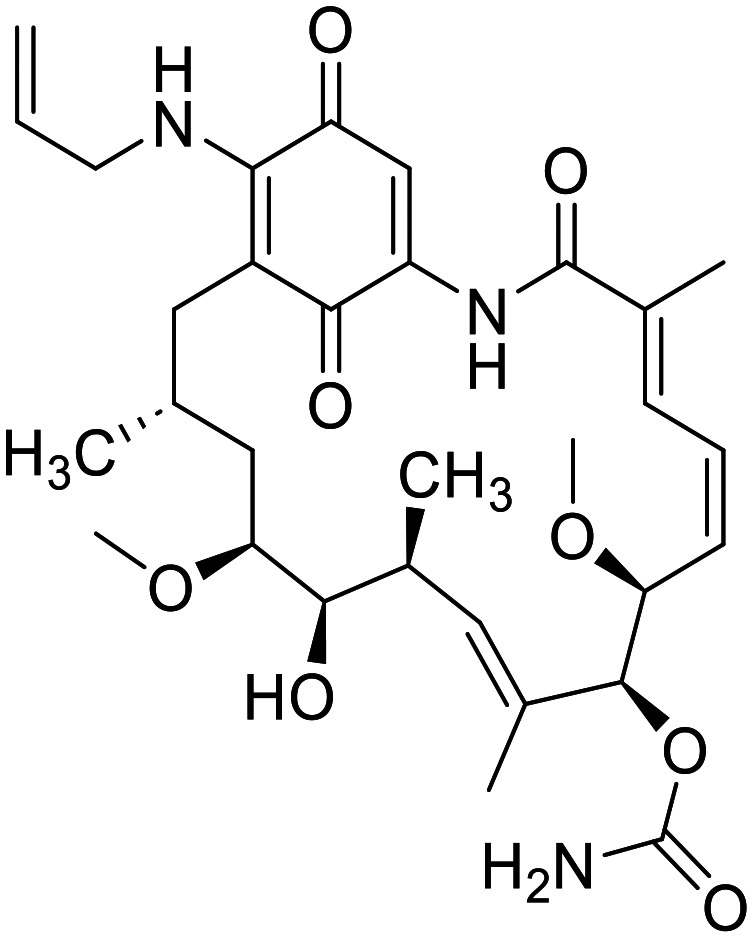	Pan-Hsp90	Alone or in combination in 38 clinical trials	Halted clinical development due to hepatotoxicity and low water solubility
17-DMAG	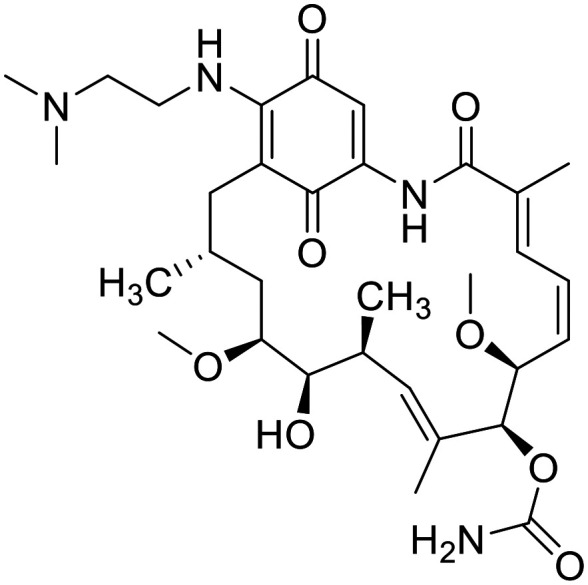	Pan-Hsp90	Alone or in combination in 7 clinical trials	Halted clinical development due to toxicity (higher compared to 17-AAG)
Ganetespib	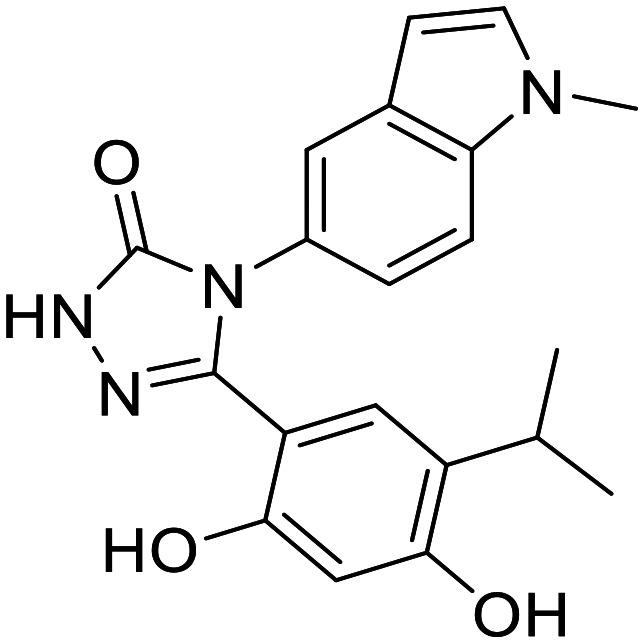	Pan-Hsp90	Alone or in combination in 38 clinical trials	24 completed, 11 withdrawn, 1 active, 2 unknown
Onalespib	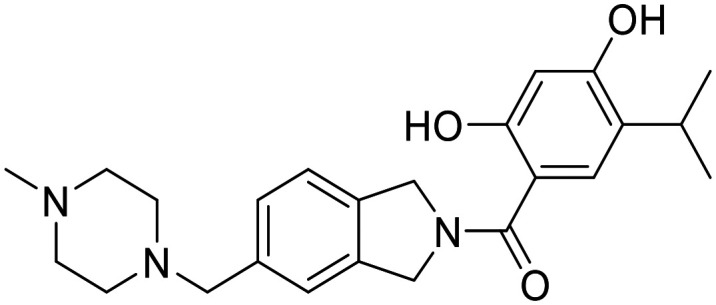	Pan-Hsp90	Alone or in combination in 13 clinical trials	6 completed, 3 withdrawn, 4 active
PU-H71	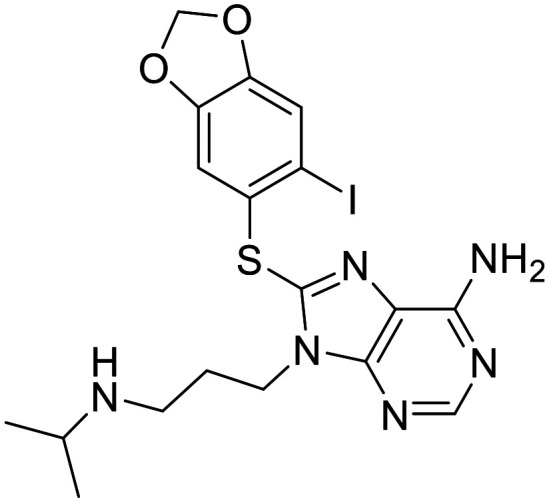	Pan-Hsp90	Alone or in combination in 6 clinical trials	3 completed or not active, 1 withdrawn, 3 active
SNX-5422	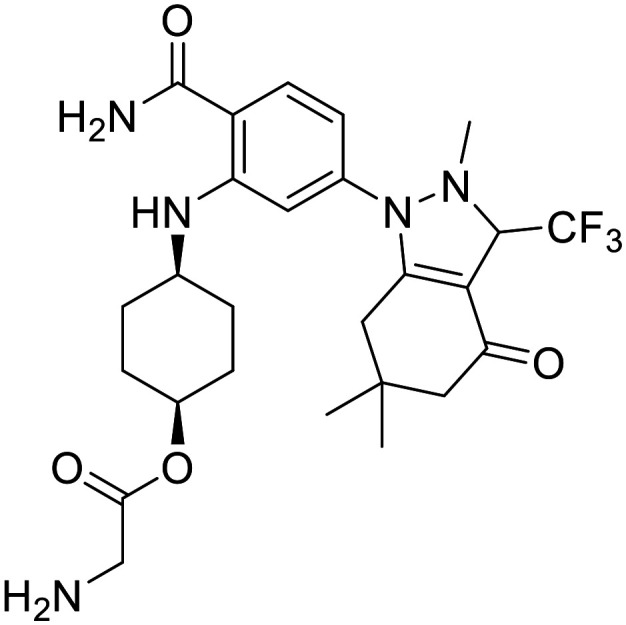	Pan-Hsp90	Alone or in combination in 6 clinical trials	Halted clinical development due to poor efficiency and financial troubles
XL888	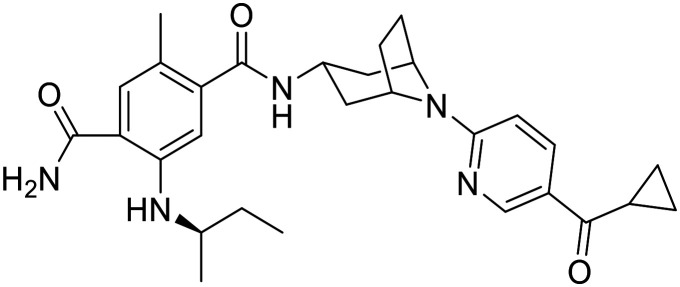	Pan-Hsp90	In combination with pembrolizumab (NCT03095781) and vemurafenib (NCT01657591)	Completed phase Ib and dose-estalation study, respectively
Gamitrinib-TPP	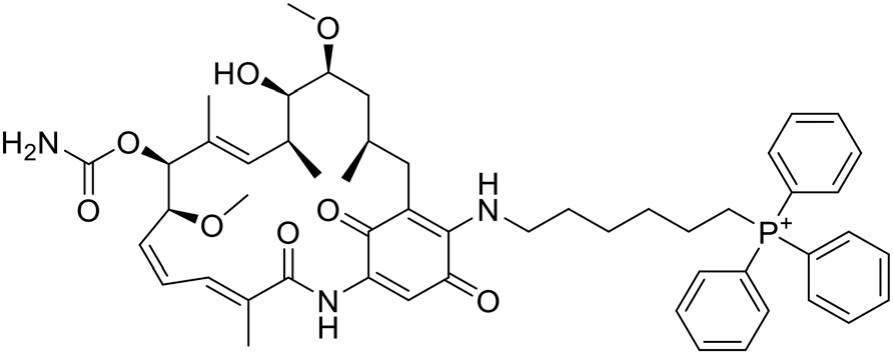	Hsp90 TRAP1	Advanced tumours (NCT04827810)	Ongoing
Luminespib	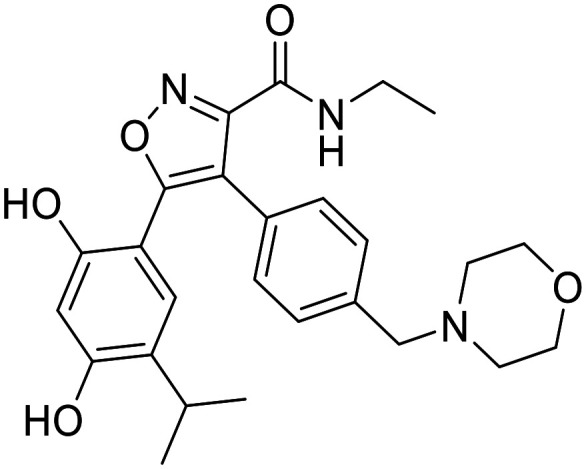	Hsp90α and Hsp90β	Alone or in combination in 27 clinical trials	Halted clinical development due to poor efficiency and limited drug availability
Pimitespib	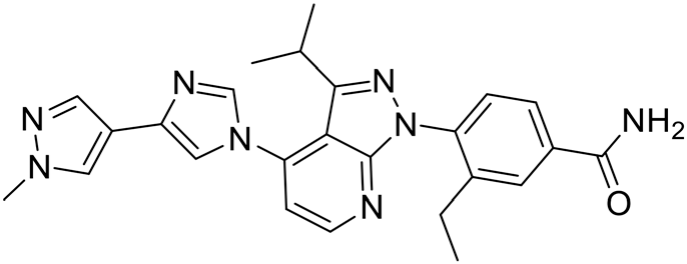	Hsp90α and Hsp90β	Alone or in combination in 4 clinical trials	1 complete, 2 recruting, 1 planned
			Used in Japan for relapsed gastrointestinal stromal tumors	

#### 3.6.1. Multitarget inhibitors

As already mentioned, multitarget inhibitors could help to overcome drug resistance and achieve synergistic anti-cancer effects. A review of structurally and functionally similar targets such as Topo II and their potential for the development of dual inhibitors has been made.^89^ The most obvious choice for the development of dual ATP-competitive inhibitors besides topoisomerase II are topoisomerase I and heat shock protein 90 (Hsp90), as they are all involved in cancer development and share structural similarities. They belong to the GHKL ATPase superfamily, which means that they have an evolutionarily conserved ATPase domain that can be exploited in the design of multitarget inhibitors.

Despite the low homology between the ATPase domains of Topo II and Hsp90, their 3D structures were compared and a pharmacophore model was constructed for the discovery of dual Topo II/Hsp90 inhibitors. The ATP binding sites of both targets were found to have similar environments. In the Topo II binding site, the Asn120 residue forms an H-bond with the adenine ring at position N6. Similarly, in Hsp90 H-bond is formed by Asp93. Ile125 forms hydrophobic interactions in the Topo II binding site that correspond to the hydrophobic Met98 in Hsp90. The pharmacophore model based on the best performing compounds docked to both targets consists of one H-bond donor site, one H-bond acceptor site and two hydrophobic regions approximately 5 Å apart that can be used for further development of dual Topo II/Hsp90 inhibitors.^127^ Another similarity was observed between Asn91 of Topo II and Asn51 of Hsp90. When compared, the orientation and configuration of the substrate (adenylyl imidodiphosphate (ANP)) in the active site were almost identical and the centre of the ATPase region was located near Mg^2+^ in both cases. Although there are no data to support quinacrine as a catalytic Topo II inhibitor or Hsp90 inhibitor, in this study it was found to inhibit the activity of both by binding to their ATP-binding pocket.^128^

Sesterterpenoid-type molecules isolated from natural marine sources, such as heteronemin, have been shown to be dual Topo II and Hsp90 inhibitors. Assays on isolated Topo II showed that heteronemin inhibits the relaxation of supercoiled DNA in a dose-dependent and catalytic manner. Docking to Hsp90 showed that hydroxy group and the oxygen of the acetyl group form H-bonds with amino acid residues in the binding site of the N-terminal domain. The 3,3-dimethylcyclohexyl ring goes into the hydrophobic cavity and forms a modest π–π interaction.^129,130^

### 3.7. Chromatin remodeling complexes inhibitiors

Chromatin remodelers are multi-subunit assemblies that can regulate DNA accessibility by modifying chromatin structure. They are driven by ATP hydrolysis, which enables the exchange, repositioning, deposition and ejection of nucleosomes and affects gene transcription. We know four subfamilies of chromatin remodeling complexes, SWItch/sucrose non-fermentable (SWI/SNF), imitation SWItch (ISWI), chromodomain helicase DNA-binding (CHD), and INO80, which differ in their composition, nucleosome interactions and effects on gene expression. They are all regularly mutated in cancer cells and represent a potential target for cancer therapies.^131^

**Fig. 10:**
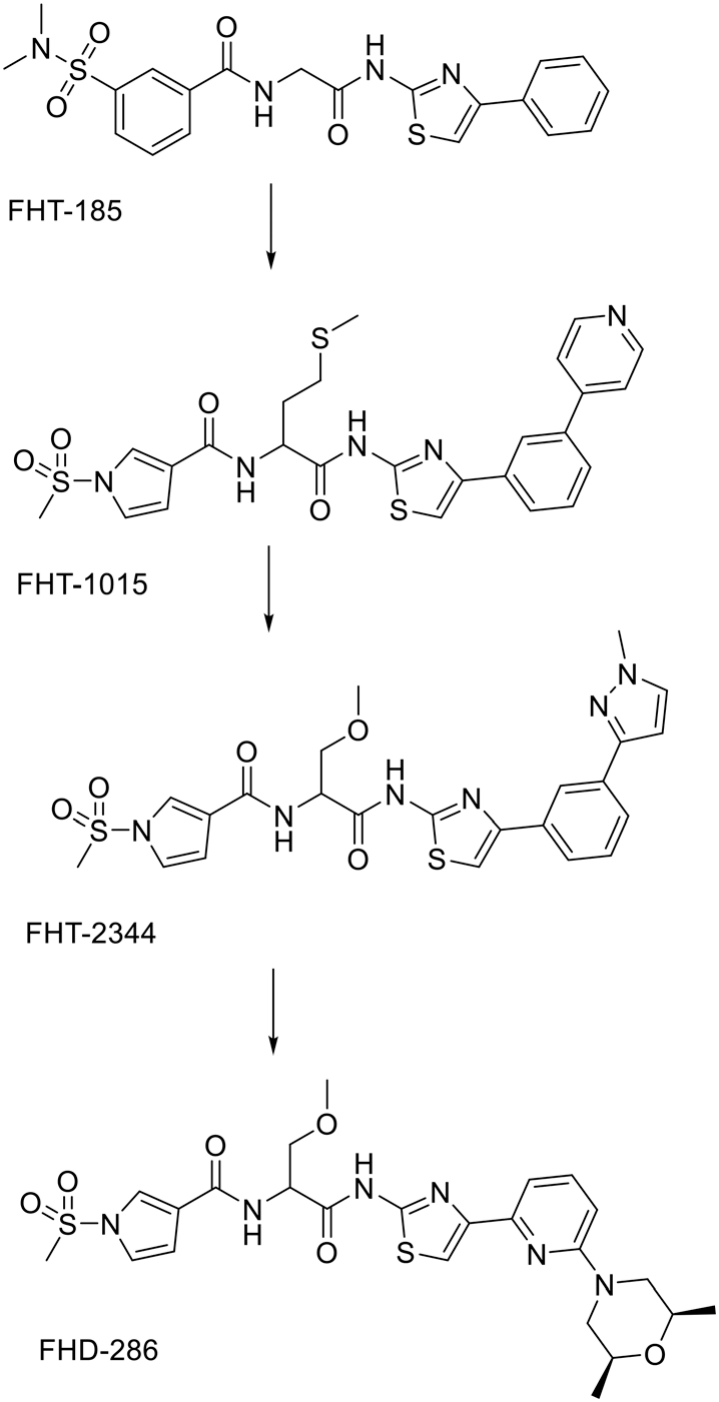
Optimization of SMARCA inhibitors.

Established SWI/SNF inhibitors inhibit its catalytic activity either by allosteric inhibition, inhibition of the bromodomain or are designed as PROTACs.^132^ There are only a number of compounds that could inhibit conserved ATPase domains located at SMARCA4/SMARCA2 subunits of SWI/SNF in an ATP-competitive manner. Initial hit, FHT-185 was optimized to FHT-1015 and ADME optimization led to the discovery of FHT-2344. FHT-2344 has low nanomolar IC_50_ values (26 nM for SMARCA4 and 13 nM for SMARCA2) and almost no inhibition of related CHD4. It was also shown to be selective for SMARCA subunits also in a cell-based assays where it was screened for binding to hundreds of ATPases. Xenograft models showed a dose-dependent inhibition of tumour growth, with no significant body weight loss observed in mice.^133^ Later, another series of dual SMARCA4/SMARCA2 inhibitors was described, with the compound FHD-286 entering phase I clinical trials for treatment of metastatic uveal melanoma (http://ClinicalTrials.gov identifier: NCT04879017). However, it was discontinued in December 2024 due to lack of efficacy,^134^ while the phase I clinical trial for the treatment of advanced hematologic malignancies is still ongoing (http://ClinicalTrials.gov identifier: NCT04891757). The *S*-enantiomer of the first screening hit was found to be responsible for the on-target activity. Optimisation of the left-hand side gave *N*-sulfonylated pyrrole residues as in FHT-2344 and optimisation of the right-hand side gave *meso*–*cis*-2,6-dimethylmorpholine. However, the resulting compound with glycine linker is poorly soluble. Replacement with an (*S*)-OMe-serine linker improved the physiochemical properties while retaining the high on-target activity of FHD-286. It showed stability in human plasma and low clearance in dog and rat liver microsomes.^135^Fig. 10 shows optimization from initial hit FHT-185 to the lead compound FHT-2344 and FHD-286.

ISWI complexes are increasingly recognised as essential for cellular survival and thus as targets in cancer therapy.^136^ They are ATP-dependent, however there are currently no known ATP-competitive inhibitors, as research is focused more on understanding ISWI's ATP-dependent remodelling mechanisms than on developing their inhibitors. SMARCA5, an ISWI catalytic subunit was proven to be inhibited by ED2-AD101. It inhibits also catalytic activity of CHD4, however ED2-AD101 acts as an allosteric rather than an ATP-competitive inhibitor.^137^

The chromodomain helicase DNA binding protein 1 like (CHD1L) is involved in the development and progression of tumours, so its inhibition represents a potential treatment option for various types of cancer. Its crystal structure (PDB: 7EPU) has been used to develop new compounds that inhibit its ATPase activity, but are thought to bind to an allosteric binding site.^138,139^ Inhibitors that bind to the ATP-binding site were discovered using virtual screening campaign. C071-0684 proved to be the most promising with an IC_50_ value of 17.36 μM in an ATPase assay and 13.0 μM on HCT116 colorectal cancer cells. Molecular docking to the ATP-binding site suggests that C071-0684 forms three hydrogen bonds and hydrophobic interactions with multiple residues within the binding site.^140^

RuvB-like 1 and 2 (RUVBL) are highly conserved AAA ATPases that are essential for many unrelated cellular processes including chromatin remodeling. They are overexpressed in several cancers, so their inhibition could lead to effective treatment.^141^ A class of pyrazolo[1,5-*a*]pyrimidine-3-carboxamide RUVBL1/2 inhibitors was discovered by docking-based screening. The most potent compound inhibited RUVBL1/2 with IC_50_ = 6.0 μM. It was the most cytotoxic on A549, HCT116, H1795, and MDA-MB-231 cell lines with IC_50_ values of 15, 11, 15 and 8.9 μM, respectively. Substituted benzyl on position 2 of pyrimidine is essential for inhibitory activity and methyl groups on positions 1 and 3 improve it significantly. Aromatic-substituted piperazinyl at position 4 is also essential for the inhibitory activity as it forms hydrophobic interactions within the binding site. Pyrazolo and pyrimidine rings form cation-π interactions and a hydrogen bond interaction and the CF_3_ group forms a potential halogen bond. When the CF_3_ group at position 3 is replaced by a CH_3_ group at position 4 and another CH_3_ group is added to the piperazine ring, the inhibition of the RUVBL1/2 complex is reduced (IC_50_ = 36 M), but inhibition of RUVBL2 is retained. This is the only compound capable of inhibiting RUVBL1 and RUVBL2 individually as well as the RUVBL1/2 complex, making it a suitable hit for further optimisation.^142^

### 3.8. Others

Other targets such as V-ATPases use ATP as an energy source for their functioning and are involved in cellular processes connected to cancer development. Interestingly, to our knowledge there are no V-ATPase inhibitors that act in an ATP-competitive manner. Promising inhibitors such as concanamycin, bafilomycin, apicularens lobatamides, benzolactone enamides *etc.* bind to the V_0_ domain, which is responsible for proton transport across membranes. ATP hydrolysis happens at the V_1_ domain, but research of V_1_ inhibitors is very limited.^143^ Analysis of the ATPase family has shown that various nucleotide-binding sites in ATPases, especially the poorly conserved adenosine-binding region where small empty cavities are present, should allow the development of ATP-competitive and selective inhibitors. So far, however, inhibitors have only been developed for enzymes with large empty cavities (Hsp90 and bacterial gyrase B). If the cavities are too small or in some cases not present at all, the development of non-ATP-competitive inhibitors is the better option.^14^

## 4. Conclusion

Cancer is a complex disease in which various cellular processes can be dysregulated. Some of the proteins involved in dysregulated processes such as Hsp90, Topo II, p97, RNA helicases, KSP, ABC transporters, chromatin remodelers, V-ATPases and protein kinases are overexpressed in cancerous cells and require ATP molecules in order to function properly. They use the chemical energy released during ATP hydrolysis as a source of energy for their work. This opens up a new way of modifying the functions of these proteins as a means of cancer therapy. In order for an ATP molecule to be hydrolysed, it must first bind to the protein in question. By developing inhibitors that compete with ATP for its binding site and cannot be hydrolysed, we can prevent the release of energy and disable the protein. Another possibility is to develop inhibitors that bind to an allosteric pocket, which causes a conformational change in the ATP-binding pocket and indirectly prevents the binding of ATP, as was the case with the KSP inhibitors. Because allosteric binding sites are not as conserved as ATP-binding pockets (particularly in members of the GHKL family), this method may provide greater specificity for the desired targets and facilitate the development of selective allosteric ATP-competitive inhibitors.

A disadvantage in development of the ATP-competitive inhibitors lies in high intracellular ATP concentrations (1–10 mM), especially in fast-dividing cancer cells. Since inhibitors compete with endogenous ATP for its binding site, their affinity for the target must be greater which requires lengthy optimization. Protein kinases have evolved to bind ATP efficiently and have high affinity for it, so kinase inhibitors must be very potent. Nevertheless, kinases are well-characterised targets and their inhibitors are developed with high ATP concentrations in mind, which has led to their clinical success over many years. Non-kinase inhibitors have not yet achieved similar success in clinical use. However, their ATP-binding sites can differ from those of kinases, making them potentially more attractive for ATP-competitive inhibitors. Conversely, this may also present new challenges, depending on the specific target.

High ATP levels are associated with phenotypically more aggressive cancer cells, their multidrug resistance, higher cell migration and metastasis and stem-like properties. There are indications that inhibitors of mitochondrial ATP-synthase could help improve therapy outcomes.^4^ They could also work synergistically with ATP-competitive compounds, as discussed high cellular concentration of ATP would be reduced.

This review provides an insight into non-kinase proteins that use ATP for their functioning and their inhibitors. Out of seven targets described, only one ATP-competitive inhibitor is being used in clinical practice (pimitespib, Hsp90). Many Hsp90 inhibitors have entered clinical trials, but have failed due to induction of heat shock response. It is a problem specific for inhibitors of N-terminal domain where the ATP-binding site is located. It can be solved with design of isoform-selective Hsp90 N-terminal inhibitors, which is currently a hot and promising research topic. Not many inhibitors targeting other proteins have entered clinical trials. CB-5339, a VCP inhibitor has proven to be the most promising as it is currently in phase I clinical trials for acute myeloid leukemia and myelodysplastic syndrome. Its ancestor, CB-5083 showed off-target effects on phosphodiesterase-6 and was retracted from clinical trials. Inhibitors for other described targets are still in the preclinical stage, but many of them show promising results. For example, ATP-competitive TopoII inhibitors address a big problem of clinically used TopoII poisons. Topo II poisons stabilize covalent TopoII-DNA complex and induce double-stranded DNA breaks that can cause chromosomal translocations and secondary malignancies within 2–3 years post treatment. ATP-competitive TopoII inhibitors act as catalytic inhibitors rather than poisons and do not induce DNA breaks, which reduces risks and makes them very promising anticancer agents. Most of the inhibitors described in this review show good on-target and cell-based activity, but require further development to enter clinical trials and obtain approval for clinical use.

Majority of them targets the ATP binding site and competes with the same ATP structure, but they are structurally quite different. Some of them mimic the structure of an ATP molecule, but form additional and stronger interactions with the binding site compared to an actual ATP molecule, making them favourable for binding to the protein of interest. Pyrazole/pyrazoline and biaryl scaffolds are common among the inhibitors studied, which is not surprising as they are found in many small molecule drugs used for various indications other than cancer. The quinazoline backbone is frequently found in p97 inhibitors. It is also found in protein kinase inhibitors already on the market. It could pose a problem for the selectivity of the developed p97 inhibitors, but selectivity for p97 has been achieved with various substituents. Selectivity towards the desired targets could also be difficult to achieve in other cases, as all the proteins studied contain an ATP-binding site. Nevertheless, the homology between the binding sites is usually low, so that sufficient structural differences exist to develop selective ATP-competitive inhibitors.

Sometimes similar binding site environments are desirable, for example in the development of dual inhibitors. In cancer treatment, it can be advantageous to treat multiple proteins simultaneously, as this can achieve synergistic effects and reduce the dose administered, which in turn reduces the severity of adverse side effects. In some cases, this may also help to overcome developed drug resistance. Some protein kinase inhibitors are known to work better due to their selectivity but not their specificity, *i.e.* they are selective for a certain group of protein kinase enzymes but not specific for only one enzyme and have a broader inhibitory effect.^144^ Some dual Hsp90 and Topo II inhibitors have been developed, either using pharmacophores or by testing compounds based on previous reports, but none of them have yet been tested in clinical trials.

Overall, the development of ATP-competitive inhibitors represents an attractive approach for cancer treatment in addition to the great success of kinase inhibitors. Various protein kinase inhibitors and several drugs targeting other ATP-dependent proteins that are already in clinical use or currently undergoing clinical trials support this claim. The fact that the ATP binding sites are similar, at least to some extent, in all ATP-dependent target proteins facilitates the development of dual inhibitors and thus the achievement of synergistic effects and the overcoming of drug resistance. We believe that ATP-competitive inhibitors will play an important role in the treatment of cancer in the future.

## Conflicts of interest

There is no conflict of interest to declare.

## Data Availability

No primary research results, software or code have been included and no new data were generated or analysed as part of this review.
